# Contents of exosomes derived from adipose tissue and their regulation on inflammation, tumors, and diabetes

**DOI:** 10.3389/fendo.2024.1374715

**Published:** 2024-08-16

**Authors:** Yanwen Wang, Qingfeng Li, Shuangbai Zhou, Pohching Tan

**Affiliations:** ^1^ Department of Plastic and Burn Surgery, West China Hospital, Sichuan University, Chengdu, China; ^2^ Department of Plastic & Reconstructive Surgery, Shanghai Ninth People’s Hospital, Shanghai Jiao Tong University School of Medicine, Shanghai, China

**Keywords:** adipose tissue, adipose-derived stem cell (ADSC), exosome, inflammation, tumor, diabetes

## Abstract

Adipose tissue (AT) serves as an energy-capacitive organ and performs functions involving paracrine- and endocrine-mediated regulation via extracellular vesicles (EVs) secretion. Exosomes, a subtype of EVs, contain various bioactive molecules with regulatory effects, such as nucleic acids, proteins, and lipids. AT-derived exosomes (AT-exos) include exosomes derived from various cells in AT, including adipocytes, adipose-derived stem cells (ADSCs), macrophages, and endothelial cells. This review aimed to comprehensively evaluate the impacts of different AT-exos on the regulation of physiological and pathological processes. The contents and functions of adipocyte-derived exosomes and ADSC-derived exosomes are compared simultaneously, highlighting their similarities and differences. The contents of AT-exos have been shown to exert complex regulatory effects on local inflammation, tumor dynamics, and insulin resistance. Significantly, differences in the cargoes of AT-exos have been observed among diabetes patients, obese individuals, and healthy individuals. These differences could be used to predict the development of diabetes mellitus and as therapeutic targets for improving insulin sensitivity and glucose tolerance. However, further research is needed to elucidate the underlying mechanisms and potential applications of AT-exos.

## Graphical Abstract

Adipose tissue comprises a variety of cells, including adipocytes, adipose-derived stem cells, macrophages, fibroblasts, and endothelial cells. These cells produce exosomes that collectively constitute adipose tissue-derived exosomes. These exosomes from adipose tissue are rich in RNA, proteins, lipids, and other components. They have diverse regulatory effects on local inflammation, adipogenesis, tumor progression, and insulin resistance levels. Furthermore, adipose tissue-derived exosomes can impact the physiological and pathological processes of muscles, skin, hypothalamus, and kidneys. (Created with BioRender.com). ADSC, adipose-derived stem cell; miRNA, microRNA; ROS, reactive oxygen species; lnc-BATE1, brown adipose tissue enriched long non-coding RNA 1; TNF-α, tumor necrosis factor-α; IL-1β, interleukin-1β; MALAT1, metastasis-associated lung adenocarcinoma transcript 1.

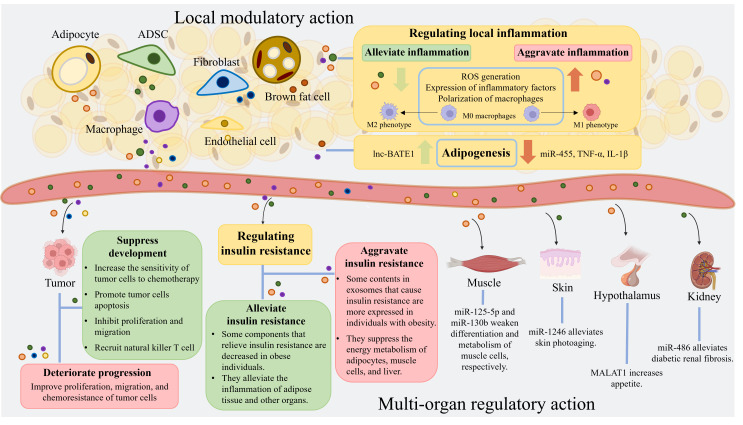

## 1 Introduction

Adipose tissue (AT) is an important energy storage organ and a vital endocrine organ that regulates the functions of other tissues and organs by secreting various signaling molecules via extracellular vesicles (EVs). These EVs affect neighboring tissues [skin (1) and muscles (2)] and distant organs (heart, lung, liver, and pancreas (3–6)). A study involving 101 patients revealed that 0.2% of serum EVs, which are derived from tissue (7), contain high levels of adipocyte proteins and adipokines (8). AT plays a specific role in the secretion of EVs harboring adipocyte proteins and adipokines, which could have systemic effects on various diseases and physiological processes.

Secretion of EVs is an important mechanism through which cells exert regulatory effects and interact with other cells and organs. EVs are categorized as exosomes, ectosomes, and apoptotic EVs based on their mode of secretion (9); this classification system differs from the previous method of categorizing EVs by size (larger EVs > 200 nm and smaller EVs < 200 nm) (10). Exosomes are formed through the inward budding of the cytomembrane (similar to endocytosis) and organelle membrane, involving the endoplasmic reticulum, Golgi apparatus, and other organelles (11). As a subtype of EVs, exosomes perform primary regulatory and therapeutic functions through their contents, including RNAs (microRNA (miRNA), long noncoding RNA (lncRNA), circular RNA, and mRNA), DNA, proteins, and lipids (12). In addition, mitochondrial components and ceramides have been identified as exosomal cargoes that participate in the regulation of the Wnt and MAPK signaling pathways (13).

**Figure 1:**
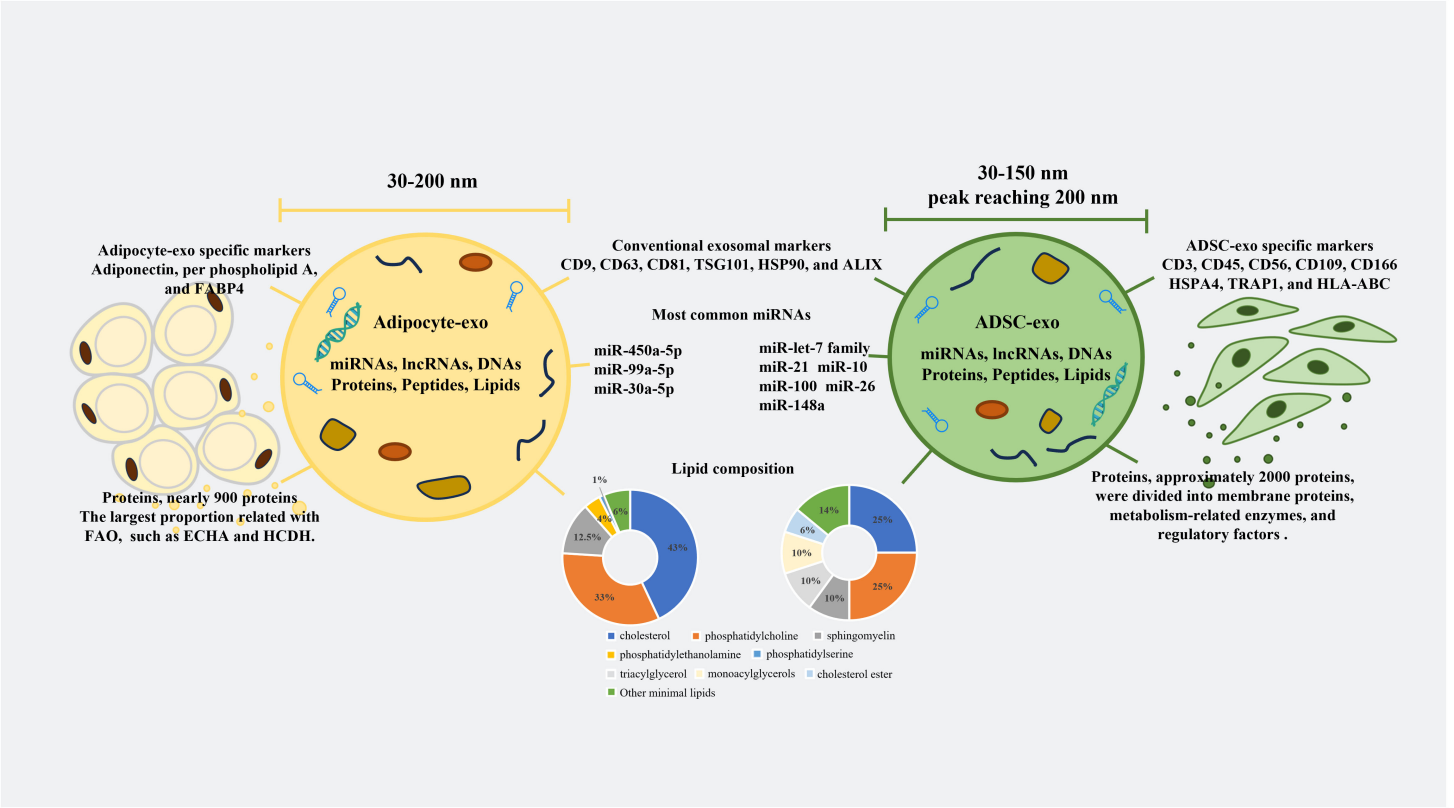
Differences between adipocyte-exos and ADSC-exos. The diameters of the adipocyte-exos were from 30 nm to 200 nm. However, the diameters of the ADSC-exos ranged from 30 nm to 150 nm, with the peak diameters reaching 200 nm. In addition to conventional exosomal markers, both adipocyte-exos and ADSC-exos have unique exosomal markers. The compositions of nucleic acids, proteins, and lipids in the adipocyte-exos and ADSC-exos were also different. FABP4, fatty acid binding protein 4; FAO, fatty acid oxidation; ECHA, trifunctional enzyme subunit alpha; HCDH, hydroxy carboxylic acid dehydrogenase; TSG101, tumor susceptibility gene 101; HSP90, heat shock protein 90; ALIX, ALG-2 interacting protein X; HSPA4, heat shock protein A4; TRAP1, tumor necrosis factor receptor-associated protein 1; HLA-ABC, human leukocyte antigen-ABC.

AT is composed of various cells, including adipocytes, adipose-derived stem cells (ADSCs), AT macrophages (ATMs), endothelial cells, progenitors, and preadipocytes. Many studies have focused on adipocyte-derived exosomes (adipocyte-exos), ADSC-derived exosomes (ADSC-exos), and ATM-derived exosomes (ATM-exos) and studied the roles of these exosomes separately. Adipocyte-exos account for a major proportion of AT-derived exosomes (AT-exos). Additionally, ADSC-exos have been shown to have meaningful therapeutic effects on multiple diseases (14–17). Extensive evidence has demonstrated that exosomes derived from ADSCs and adipocytes can regulate localized inflammation and metabolic diseases (3, 17), promote wound healing (18, 19), and affect tumor growth and migration (20, 21). However, ADSC-exos are significantly more effective at promoting angiogenesis and protecting the heart and blood vessels than adipocyte-exos. The diameters, different surface features, and major RNAs and lipid components of adipocyte-exos and ADSC-exos are summarized in **Figure 1**. The majority of proteins in adipocyte-exos are mitochondrial fatty acid oxidase enzymes, such as trifunctional enzyme subunit alpha and hydroxyacyl-coenzyme A dehydrogenase (22). ADSC-exos contain various growth factors and enzymes associated with glycolysis, such as phosphoglucomutase, phosphoglycerate kinase, glyceraldehyde 3-phosphate dehydrogenase, enolase, and pyruvate kinase m2 isoform (23), as well as enzymes with dephosphorylation functions (24). Moreover, ATM-exos can play a regulatory role in insulin resistance (25). Furthermore, all of the cells mentioned above produce exosomes, which constitute AT-exos. In summary, AT-exos is complex.

AT-exos potentially exert regulatory and therapeutic effects on numerous diseases, such as diabetic wounds (19), insulin resistance (26), angiogenesis (27), and nonalcoholic steatohepatitis (28). Different components in AT-exos may regulate the same disease or physiological process in various directions. For different populations, the cargoes delivered by AT-exos and their regulatory effects can vary greatly. For instance, the quantity of AT-exos, especially adipocyte-exos, in obese individuals is significantly increased (29). Moreover, substances within adipocyte-exos, ADSC-exos, and ATM-exos that can alleviate tissue inflammation and mitigate insulin resistance are markedly reduced (30), while those that exacerbate tissue inflammation and worsen insulin resistance are noticeably increased in adipocyte-exos and ATM-exos (31). Consequently, the AT-exos in obese individuals significantly intensify inflammation in local AT and lead to systemic insulin resistance. Because AT-exos is complex, focusing on the effects of AT-exos will provide a more comprehensive and objective understanding than studying one type of cell-derived exosomes in isolation when studying the impact of AT on other tissues and organs.

## 2 The regulation of inflammation by exosomes derived from AT in the local microenvironment

In the local microenvironment, adipocyte-exos, ADSC-exos, and ATM-exos carry multiple miRNAs and proteins that participate in regulating inflammation. Among these cargoes, some RNAs and proteins can alleviate inflammation, while others can exacerbate inflammation. Notably, the ability of AT-exos to regulate inflammation varies among different populations. AT-exos from obese and aged individuals are more likely to exacerbate inflammation (17, 32).

### 2.1 The influence of RNAs derived from AT-exos on regulating inflammation

Some RNAs in adipocyte-exos and ADSC-exos can alleviate inflammation in the local microenvironment. Nine adipocyte-exo-derived miRNAs (miR-26a, miR-92a (33), miR-126, miR-143, miR-193a, miR-193b, miR-652, miR-let-7a, and miR-let-7d) can repress the production of C-C motif ligand 2 (CCL2), which induces the polarization of macrophages to the proinflammatory phenotype and is present in higher levels in the serum of obese individuals than in lean individuals (34). miRNAs in ADSC-exos exert a significant effect on inducing M2 macrophage polarization. Activation of NOD-like receptor protein 3 was also proven to be suppressed by ADSC-derived EVs in previous studies (35, 36). In addition, compared to those in old mice, the ADSC-exos in young mice contained higher levels of miR-125b-5p, miR-214-3p, and miR-let7c-5p, which may explain the decreased expression of inflammatory markers and the regeneration of muscle and kidney in old mice injected with ADSC-exos from young mice (17).

AT-exos, including both adipocyte-exos and ADSC-exos, also contain miRNAs that exacerbate inflammation in the AT microenvironment. Moreover, a study confirmed that ADSC-exos also promote macrophage infiltration by upregulating the levels of monocyte chemoattractant protein-1 and macrophage inflammatory protein-1α in a fat transfer experiment (37).

### 2.2 The effects of exosomal proteins and lipids on the regulation of inflammation

The proteins in adipocyte-exos that are implicated in the regulation of inflammation include adipokines (adiponectin, leptin, resistin, and autotaxin), cytokines (interleukin (IL)-6, IL-1β, IL-8, CCL2, CCL5, and TNF-α), and adipsin (38, 39). Among the proteins in adipocyte-exos, adiponectin significantly affects anti-inflammation and is present in higher levels in the AT space and plasma of lean individuals (40). These cytokines in adipocyte-exos have apparent proinflammatory effects (40). For ADSCs, ADSC-exos carry proinflammatory cytokines (IL-1β, IL-7, IL-8, IL-9, IL-11, IL-12, IL-15, IL-17, IFN-γ, and TNF-α) and anti-inflammatory factors (IL-1Ra, IL-4, IL-10, and IL-13) (41), while IL-2, IL-6, and adipsin have both proinflammatory and anti-inflammatory characteristics (42, 43).

Upon the stimulation of M1 polarization, compared with adiponectin-negative EVs, adiponectin-positive EVs derived from adipocytes promote monocyte differentiation into ATM through the secretion of TNF-α, macrophage colony-stimulating factor, and retinol-binding protein 4 (44). A separate study indicated that retinol-binding protein 4 in adipocyte-exos facilitates the M1 polarization of monocytes and the production of proinflammatory cytokines. Furthermore, levels of exosomal retinol-binding protein 4 are elevated in obese mice (30).

The functional RNAs, proteins, and lipids in exosomes derived from AT that regulate the inflammation of the microenvironment are listed in **Table 1**.

**Table 1:** RNAs, proteins, and lipids in exosomes derived from AT regulate inflammation.

Substances	Derived	Function	Mechanisms	References
lncRNA-SNHG9	Adipocyte	Alleviate inflammation	lncRNA-SNHG9 binds with the TRADD mRNA and silences TRADD to reduce the expression of inflammatory factors in endothelial cells and prevent the apoptosis of endothelial cells.	(45, 46)
miR-17-5p	ADSC	Alleviate inflammation	miR-17-5p binds to the 3′-untranslated region of TXNIP and decreases the expression of the TXNIP-NLRP3 signaling pathway in the macrophages.	(47)
miR-27b-3p	ADSC	Alleviate inflammation	miR-27b-3p binds to the CSF-1 mRNA to inhibit its translation, causing the polarization of monocytes to M2 macrophages and alleviating inflammation.	(48)
miR-451a	ADSC	Alleviate inflammation	miR-451a targets macrophage migration inhibitory factor to promote the M2 polarization and relieve the inflammation in bone healing.	(49)
miR-1931	ADSC	Alleviate inflammation	miR-1931 targets TRAF6, the transducer of the NF-κB pathway, and inhibits the NF-κB signal to suppress the M1 macrophage polarization.	(50)
miR-223	Adipocyte and ADSC	Alleviate inflammation	miR-223 targets and reduces NLRP3, an important component of the inflammasome, to relieve the severity of inflammation.	(51–53)
miR-155	Adipocyte	Aggravate inflammation	miR-155 targets SOCS1 and induces M1-polarization by activating the expression of STAT1 and inhibiting STAT6 expression.	(54)
miR-34a	Adipocyte	Aggravate inflammation	miR-34a represses the expression of KLF4 to decrease the differentiation of macrophages to M2 polarization. Furthermore, miR-34a in VAT is enriched selectively more than that in the SAT, with a higher expression of miR-34a in VAT after feeding on the HFD. The greater enrichment of miR-34a in VAT can potentially elucidate why VAT is more susceptible to inflammation compared to SAT.	(3, 4)
Adiponectin	Adipocyte	Alleviate inflammation	(1) Adiponectin induces macrophages to polarize to M2 macrophage phenotype, with higher expression of arginase-1 and IL-10. (2) Adiponectin reduces the production of ROS via the NADPH oxidase and suppresses the activity of TNF-α in the macrophages. (3) Adiponectin can decrease the chemokine CXCL8 production in the peripheral blood neutrophils. (4) Adiponectin promotes the formation of APPL1/leptin complex, activating TCF/LEF and Wnt/β-catenin signaling to upregulate the expression of CD44 in the vascular tissue.	(55–58)
AdipoAI	Adipocyte	Alleviate inflammation	AdipoAI, an adiponectin receptor agonist, reduces the expression of pro-inflammatory cytokines in LPS-induced macrophages, with the effect on myeloid differentiation marker 88 signaling to attenuate the association of the adiponectin receptors and inhibits the activation of NF-κB, MAPK, and c-Maf pathways.	(59)
α-ketoglutarate	Adipocyte	Alleviate inflammation	α-ketoglutarate can attenuate STAT3/NF-κB signal in adipocytes and induce the M2 polarization.	(60)
STAT3	ADSC	Alleviate inflammation	STAT3 activates the transcription of arginase-1 to promote M2 macrophage polarization after internalizing in macrophages.	(61)
18 hydroxy-eicosatetraenoic acid	ATM	Alleviate inflammation	18 hydroxy-eicosatetraenoic acid inhibits macrophage-mediated pro-inflammatory reactions and alleviates the cardiac fibroblasts.	(62)
Leptin	Adipocyte	Aggravate inflammation	Leptin upregulates ROS production and inhibits the migration of the neutrophils. Leptin can increase the expression of inflammatory factors (IL-2, IL-12, and IFN-γ) in monocytes to induce a Th1-dominant immune response in infection.	(40, 63)
Autotaxin	Adipocyte	Aggravate inflammation	Autotaxin acts on LPA receptors to stimulate multiple G-protein mediated signal transduction pathways, including the Enpp2 gene, Ras/Raf/MEK/ERK pathway, and PI3K signaling pathway, and causes more inflammation in fibroblasts, epithelial cells, and endothelial cells, resulting in higher levels of IL-8 and TNF-β to aggravate the tissue fibrosis.	(64)
TNF-α and IL-1β	ATM	Aggravate inflammation	TNF-α and IL-1β repress the adipogenesis of ADSC and combine with IFN-γ to promote the immunosuppressive properties of ADSC by excreting more indoleamine 2,3-dioxide in humans or more iNOS in mice.	(41, 65, 66)
Resistin	ATM in humans and adipocytes in mice	Aggravate inflammation	Resistin exacerbates insulin resistance and inflammatory metabolic diseases in adipocytes, hepatocytes, myocytes, endothelial cells, and other cells by influencing the pattern recognition receptor TLR4.	(67–69)
Adipsin	Adipocyte	Both alleviate and aggravate inflammation	Adipsin removes the dead cells to alleviate inflammation and promotes the production of chemotactic factors to exacerbate inflammation.	(43)

lncRNA-SNHG9, lncRNA-small nucleolar RNA host gene 9; TRADD, TNF receptor type 1-associated death domain protein; mRNA, messenger RNA; TXNIP, thioredoxin-interacting protein; NLRP3, NOD-like receptor protein 3; CSF-1, colony stimulating factor-1; TRAF6, tumor necrosis factor receptor-associated factor 6; NF-κB, nuclear factor kappa-B; SOCS1, suppressor of cytokine signaling 1; STAT1, signal transducer and activator of transcription 1; STAT6, signal transducer and activator of transcription 6; KLF4, Krüppel-like factor 4; VAT, visceral adipose tissue; SAT, subcutaneous adipose tissue; HFD, high-fat diet; ROS, reactive oxygen species; NADPH, nicotinamide adenine dinucleotide phosphate; TNF-α, tumor necrosis factor-α; CXCL8, C-X-C motif ligand 8; APPL1, adaptor protein containing PH domain, PTB domain, and leucine zipper motif 1; TCF, T-cell factor; LEF, lymphoid enhancer-binding factor; Wnt, wingless/integrated; AdipoAI, adipo anti-inflammation agonist; LPS, lipopolysaccharide; MAPK, mitogen-activated protein kinases; STAT3, signal transducer and activator of transcription 3; IL-2, interleukin-2; IL-12, interleukin-12; IFN-γ, interferon-γ; LPA, lysophosphatidic acid; Enpp2, ecto-nucleotide pyrophosphatase/phosphodiesterase family member 2; Ras, rat sarcoma; Raf, rapidly accelerated fibrosarcoma; MEK, mitogen-activated protein kinase kinase; ERK, extracellular signal-regulated kinases; PI3K, phosphoinositide 3-kinase; IL-8, interleukin-8; TNF-β, tumor necrosis factor-β; IL-1β, interleukin-1β; iNOS, inducible nitric oxide synthase; TLR4, Toll-like receptor 4.

## 3 The regulatory effect of exosomes derived from AT on tumors

**Figure 2:**
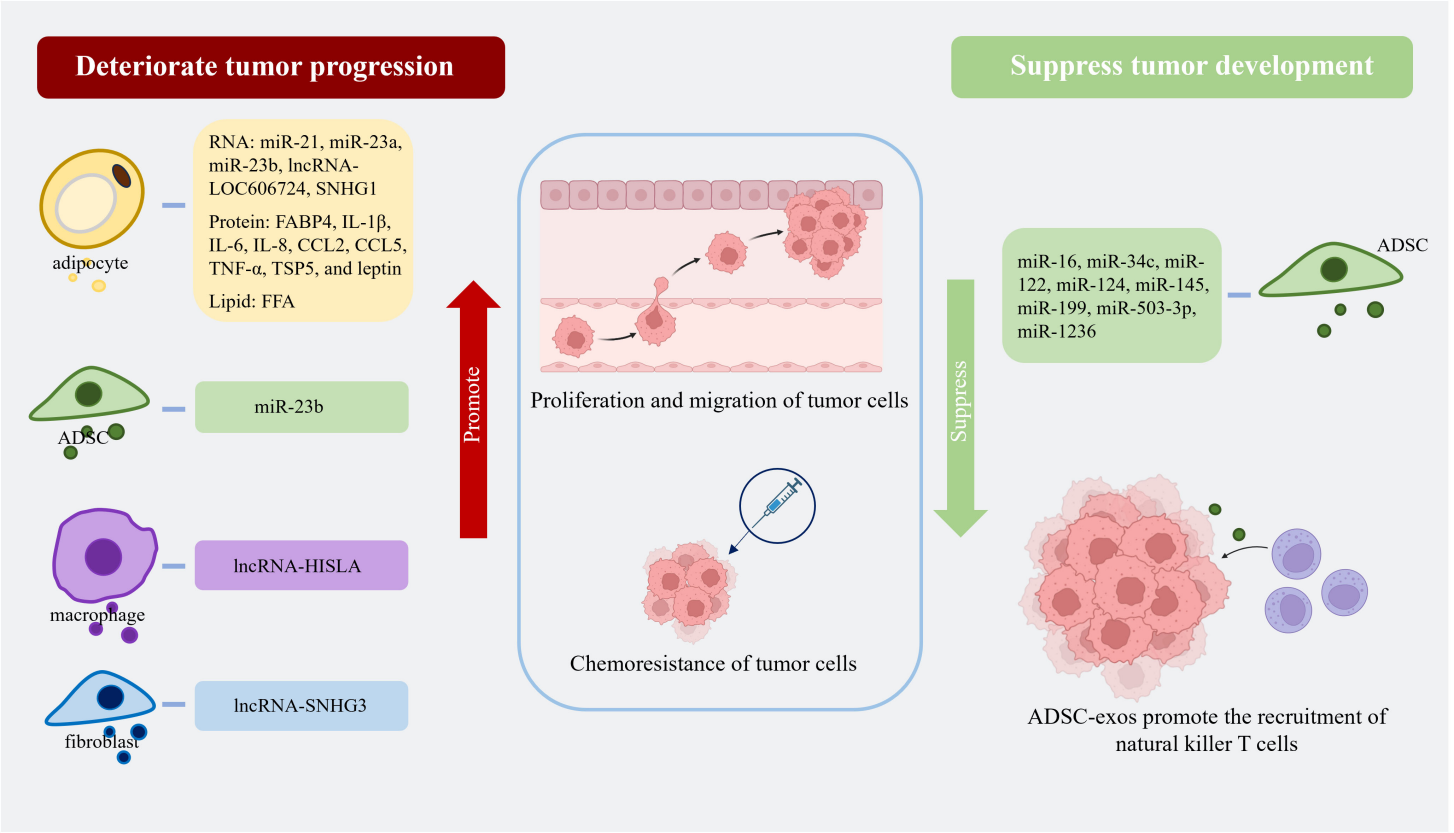
Adipocyte-exos, ADSC-exos, ATM-exos, and fibroblast-derived exosomes all contain substances that can promote tumor progression by enhancing the proliferation, migration, and chemoresistance of tumor cells. ADSC-exos are rich in various miRNAs that can suppress tumor growth by increasing the sensitivity of tumor cells to chemotherapy drugs, promoting apoptosis, inhibiting proliferation and migration, and recruiting natural killer T cells. (Created with BioRender.com). lncRNA-SNHG1, lncRNA-small nucleolar RNA host gene 1; FABP4, fatty acid binding protein 4; IL-1β, interleukin-1β; IL-6, interleukin-6; IL-8, interleukin-8; CCL2, C-C motif ligand 2; CCL5, C-C motif ligand 5; TNF-α, tumor necrosis factor-α; TSP5, thrombospondin family protein 5; FFA, free fatty acid; lncRNA-HISLA, lncRNA-HIF-1α-stabilizing long noncoding RNA; lncRNA-SNHG3, lncRNA-small nucleolar RNA host gene 3.

AT-exos, including adipocyte-exos, ADSC-exos, ATM-exos, endothelial cell-derived exosomes, and exosomes from other AT-derived cells, can affect both adjacent and distant tumor cells. To promote tumor growth, adipocyte-exos can be transported into adjacent tumor cells, such as breast cancer (BC) cells, and improve the proliferation, migration, and chemoresistance of BC cells; this effect is related to stimulating the expression of YAP and TAZ, which are two key downstream proteins of the Hippo signaling pathway (70). In a study on triple-negative BC, cancer-associated adipocytes deliver more C-X-C motif ligand 8 to adjacent BC cells compared to normal adipocytes. C-X-C motif ligand 8 upregulates the expression of the PI3K/AKT/mTOR pathway in tumor cells, promotes the proliferation and epithelial-mesenchymal transition of tumor cells, and enhances the expression of PD-L1 on the BC cell membrane, which is detrimental to the long-term prognosis of patients (71). Fatty acid binding protein 4 derived from adipocyte-exos, expressed at higher levels in metastatic ovarian cancer patients than in primary ovarian cancer patients, is considered a marker of metastatic tumor disease and a therapeutic target (72). Some miRNAs, such as let-7-a-1, miR-21, and miR-1260b, are significantly enriched in ADSC-exos from patients with cancer (73). Moreover, ADSC-exos promote tumor progression by facilitating the proliferation and migration of cancer cells. In comparison to those in the control group, ADSC-exos activates the Wnt signaling pathway in the MCF7 BC cell line and promotes the migration of cancer cells (74). Fewer microvesicles originating from the endothelial cells of BC patients are implicated in better clinical outcomes after chemotherapy (75). Endothelial cell-derived exosomes may also contribute to cancer progression. On the unfavorable aspects of tumors, ADSC-exo-derived miRNAs can suppress tumor growth by increasing the sensitivity of cancer cells to chemotherapy, reducing the expression of drug-resistance genes, promoting cancer cell apoptosis, inhibiting the tumor proliferation and migration, and recruiting natural killer T cells. ADSC-exos can recruit natural killer T cells and promote their antitumor responses in the tumor microenvironment. In an N1S1-induced hepatocellular carcinoma model, mice treated with ADSC-exos had markedly smaller tumor volumes and more circulating and intratumoral natural killer T cells than the control mice (76). In brief, AT-exos can suppress tumor development and promote the proliferation, migration, and drug resistance of tumor cells through the delivery of miRNAs, proteins, and lipids. The contents in various AT-exos affecting tumor progression are summarized in **Figure 2**. The mechanisms underlying their effects are presented in **Table 2**.

**Table 2:** Effective substances in exosomes derived from AT impact the tumor progression.

Deteriorate tumor progression				
Substance		Derived	Mechanisms	References
RNA	miR-21	Adipocyte and ADSC	miR-21 reduces the expression of the tumor suppressor gene PTEN and promotes the differentiation of macrophages into TAMs.	(77, 78)
	miR-23a and miR-23b	Adipocyte miR-23b also found in ADSC-exo	miR-23a and miR-23b target the von Hippel-Lindau/hypoxia-inducible factor axis to promote chemoresistance of HCC cells and facilitate the growth and metastasis of HCC. miR-23b can target myristoylated alanine-rich C-kinase substrate in BC cells to regulate the cell cycle, alter their dormant state, and protect cancer cells from the cytotoxic effects of treatments.	(20, 79, 80)
	lncRNA-SNHG3	Cancer-associated fibroblasts	lncRNA-SNHG3 represses miR-330-5p and upregulates Pyruvate Kinase M1/M2 expression to promote the glycolysis and the proliferation of BC cells.	(81)
	lncRNA-HISLA	TAM	lncRNA-HISLA combines with prolyl hydroxylase domain 2 which targets HIF-1α to repress its hydroxylation and degradation, enhancing aerobic glycolysis and apoptosis resistance of BC cells. Meanwhile, BC cells can release lactate to increase the expression of HISLA in TAM, forming a synergistic closed loop between TAMs and cancer cells.	(82, 83)
	lncRNAs, LOC606724 and SNHG1	MM-associated adipocytes	These two lncRNAs are abundant in MM cells and develop the chemoresistance of MM cells. And MM cells can improve the levels of these two lncRNAs in adipocyte-exo by methyltransferases such as 7A which is an RNA methyltransferase and mediates lncRNA m^6^A methylation. This process results in a loop to enhance the resistance of MM cells to chemotherapy between MM cells and MM-associated adipocytes.	(84)
Protein	FABP4	Adipocyte	FABP4 transfers the fatty acids from adipocytes to tumor cells and accelerates proliferation and angiogenesis in cancer.	(85)
	IL-1β, IL-6, IL-8, CCL2, CCL5, and TNF-α	Adipocyte	These cytokines contribute to the proliferation and migration of cancer cells via the activation of MAPK/ERK, JAK2/STAT3, and PI3K/AKT signaling pathways.	(39, 86, 87)
	TSP5	Adipocyte	TSP5 induces the upregulation of EMT genes, such as BRD2 and BRD4, in recipient cells, leading to the occurrence of BC easily.	(88)
	Leptin	Adipocyte	Leptin targets the Ob-R/LEPR receptor on the cancer cell surface to enhance the expression of PLOD2 and activates signaling pathways, such as JAK/STAT3 and PI3K/AKT signaling pathways, as well as the adipocyte-derived IL-6. Leptin contributes to the maintenance of androgen phenotype in BC, the cancer cell proliferation, and the EMT in ovarian cancer cells.	(89–91)
Lipid	FFA	Adipocyte	FFA supplies energy, affects biosynthesis, and regulates the endocrine system. FFA can also activate the PPARα signaling pathway to upregulate the expression of catabolic enzymes in hepatocytes and accelerate the progress of FAO in the mitochondria to enhance cancer cell migration in melanoma cells.	(22, 92)
Lactic acid and glutamic acid		Bone marrow mesenchymal stem cell	Lactic acid and glutamic acid are the energy-supplying substance and the raw material for substance synthesis, respectively.	(93)
Suppress tumor development. (All the following miRNAs were derived from ADSC-exo)				
miRNA		Mechanisms		Reference
miR-34c		miR-34c targets and silences β-catenin to promote radiation-induced apoptosis in NPC cells. miR-34c inhibits the malignant behavior of NPC, such as proliferation, migration, invasion, and epithelial-mesenchymal transition.		(21, 94)
miR-122		miR-122 inhibits the expression of target genes CCNG1, ADAM10, and IGF1R, and increases the sensitization of HCC to 5-FU and sorafenib.		(95)
miR-124 and miR-145		miR-124 and miR-145 inhibit the migration of glioma cells and self-renewal of glioma stem cells by targeting SCP-1 and SOX2, relatively. miR-124-3p is reported to suppress glioma proliferation through the FLOT2/AKT1 pathway. miR-145 can reduce the activity of Bcl-X(L) in prostate cancer cells, thereby suppressing cell proliferation and promoting tumor apoptosis.		(96–98)
miR-199		miR-199 can downregulate the expression of the mTOR signaling pathway to enhance the sensitivity of HCC to DOX. miR-199 also targets the AGAP2 gene and suppresses its expression, enhancing the sensitivity of glioma cells to temozolomide and inhibiting tumor progression.		(99, 100)
miR-503-3p		miR-503-3p restrains the proliferation and self-renewal of cancer stem cells.		(101)
miR-1236		miR-1236 targets SLC9A1 and represses the Wnt/β-Catenin signaling pathway.		(102)

PTEN, phosphatase and tensin homolog; TAM, tumor-associated macrophage; HCC, hepatocellular carcinoma; BC, breast cancer; lncRNA-SNHG3, lncRNA-small nucleolar RNA host gene 3; lncRNA-HISLA, lncRNA-HIF-1α-stabilizing long noncoding RNA; HIF-1α, hypoxia-inducible factor-1α; lncRNA-SNHG1, lncRNA-small nucleolar RNA host gene 1;MM, multiple myeloma; IL-1β, interleukin-1β; IL-6, interleukin-6; IL-8, interleukin-8; CCL2, C-C motif ligand 2; CCL5, C-C motif ligand 5; TNF-α, tumor necrosis factor-α; MAPK, mitogen-activated protein kinases; ERK, extracellular signal-regulated kinases; JAK2, Janus Kinase 2; STAT3, signal transducer and activator of transcription 3; PI3K, phosphoinositide 3-kinase; AKT, protein kinase B; TSP5, thrombospondin family protein 5; EMT, epithelial-mesenchymal transition; BRD2, bromodomain-containing protein 2; BRD4, bromodomain-containing protein 4; Ob-R/LEPR, leptin receptor; PLOD2, procollagen-lysine 2-oxoglutarate 5-dioxygenase 2; FFA, free fatty acid; PPARα, peroxisome proliferator-activated receptor-α; FAO, fatty acid oxidase; NPC, nasopharyngeal carcinoma; CCNG1, cyclin G1; ADAM10, a disintegrin and metalloproteinase 10; IGF1R, insulin like growth factor 1 receptor; 5-FU, 5-fluorouracil; SCP-1, stem cell protein-1; SOX2, SRY (sex determining region Y)-box 2; FLOT2, flotillin-2; Bcl-X(L), B-cell lymphoma-extra large; mTOR, mechanistic target of rapamycin; DOX, doxorubicin; AGAP2, ArfGAP with GTPase domain, Ankyrin repeat and PH domain 2; SLC9A1, solute carrier family 9 member A1.

## 4 The effects of exosomes derived from AT on obesity and insulin resistance

As the most important energy storage organ in the human body, AT also performs strong endocrine regulatory functions. AT-exos have been proven to play a regulatory role in the sensitivity of the body to insulin through multiple signaling pathways and are strongly correlated with individual obesity.

Compared to those in healthy and lean individuals, AT-exos in the obese population exhibit significant differences. First, in terms of quantity, the AT of obese individuals and patients with insulin resistance can produce more exosomes (103). Ceramide can promote the membrane curvature of EVs and exert a significant effect on the vesicle budding process. Inhibiting ceramide production can reduce the biogenesis of EVs (104). Moreover, the budding process is influenced by palmitic acid and phospholipase D (105). Obese individuals have a greater variety of ceramides in AT (106) and excessive palmitic acid in enlarged adipocytes (107), which are conducive to the budding of multivesicular bodies in adipocytes, especially in obese individuals. Furthermore, the increased production of exosomes in AT of obese individuals mainly occurs due to increased production by adipocytes, with no significant increase by the other types of cells (108). The number of adipocyte-exos in the circulation of obese mice was approximately twofold higher than that in lean mice (29). Chronic mild inflammation, which is a biological stimulus of AT associated with obesity, might facilitate the secretion of adipocyte-exos (109). However, increased adipocyte-exos could accelerate the excretion of harmful cytoplasmic substances and prevent cellular senescence. The contents and cargoes of adipocyte-exos can also impact other cells and organs throughout the body through paracrine or endocrine effects. The implications of this phenomenon on the human body are intricate and cannot be conclusively defined as beneficial or detrimental. Moreover, the mechanism underlying the increased production of adipocyte-exos in obesity remains incompletely understood (110). The expression profiles of AT-exos, including the levels of miRNAs, proteins, and lipids, also vary between obese individuals and healthy individuals.

### 4.1 Differentially expressed miRNAs in obese and healthy individuals

As previously mentioned, the following miRNAs are upregulated in the adipocyte-exos of obese individuals: miR-23b, miR-27a, miR-99b, miR-122, miR-140-5p, miR-142-3p, miR-192, miR-222, miR-378a, and miR-4429 (3, 111–114). Moreover, the expression of miR-15a, miR-26a, miR-30c, miR-92a, miR-126, miR-130b, miR-138, miR-143, miR-145, miR-148b, miR-193a, miR-193b, miR-221, miR-223, miR-423-5p, miR-520c-3p, miR-652, miR-4269, miR-let-7af, and miR-let-7d is significantly decreased in the adipocyte-exos of obese individuals (31, 34, 115–119). Among the decreased miRNAs, nine could inhibit the expression of CCL2 to suppress the M1 polarization of macrophages and alleviate inflammation when expressed at higher levels in healthy individuals (34). The influence and working mechanisms of these differential miRNAs are summarized in **Table 3**.

**Table 3:** The miRNAs in exosomes derived from AT with different expressions are related to insulin resistance and obesity.

miRNA	Derived	Function	Mechanisms	Reference
miR-27a	Adipocyte	Aggravate insulin resistance	miR-27a suppresses PPARγ in skeletal muscle cells to cause insulin resistance.	(118)
miR-99b	Adipocyte	Aggravate insulin resistance	miR-99b can bind to the mRNA of FGF21 in liver cells, influencing liver metabolism and exacerbating insulin resistance and glucose tolerance.	(3, 120)
miR-155	Adipocyte and ATM	Aggravate insulin resistance	miR-155 derived from adipocyte-exo targets SOCS1, leading to the upregulation of STAT1 and downregulation of STAT6, and promoting M1 macrophage polarization. M1 macrophages can inhibit the phosphorylation of AKT by insulin and reduce glucose uptake in adipocytes. miR-155 derived from ATM-exo downregulates adipogenic transcription factors, PPARγ and CEBPβ, leading to insulin resistance and hindering hepatic glucose output. In pancreatic β cells, inhibition of v-maf musculoaponeurotic fibrosarcoma oncogene family protein B by miR-155 can reduce insulin secretion.	(54, 121, 122)
miR-222	Adipocyte	Aggravate insulin resistance	miR-222 targets Glut-4 to regulate glucose uptake in adipocytes and inhibits the expression of insulin receptor substrate-1 in hepatocytes. Its levels are elevated in both the blood and fat tissue of obese individuals.	(111, 112)
miR-126	Adipocyte and ADSC	Alleviate insulin resistance	miR-126 benefits the promotion of angiogenesis and reduces inflammation in AT, with lower expression in obese individuals.	(32, 123)
miR-223	Adipocyte and ADSC	Alleviate insulin resistance	miR-223 targets NLRP3 and alleviates the inflammation in AT. miR-223 can also bind to HDL in the circulation. It is decreased in the blood of T2DM patients.	(35, 36, 119, 124)

PPARγ, peroxisome proliferator-activated receptor-γ; mRNA, messenger RNA; FGF21, fibroblast growth factor 21; SOCS1, suppressor of cytokine signaling 1; STAT1, signal transducer and activator of transcription 1; STAT6, signal transducer and activator of transcription 6; AKT, protein kinase B; CEBPβ, CCAAT/enhancer-binding protein β; Glut-4, glucose transporter 4; NLRP3, NOD-like receptor protein 3; HDL, high-density lipoprotein; T2DM, type 2 diabetes mellitus.

Taken together, these data show that the aberrant expression of these miRNAs in obese individuals could worsen inflammation in AT, leading to obesity. Several plasma miRNAs derived from adipocytes could be considered predictive factors of type 2 diabetes mellitus. For example, increased miR-15b levels and decreased miR-138 levels could be considered characteristic of obese individuals compared with individuals in the normal control group (125, 126). Furthermore, a combined analysis of circulating miR-15a, miR-423-5p, and miR-520c-3p, which are downregulated in obese individuals, could predict whether a man has morbid obesity with an accuracy of up to 93.5% (115).

### 4.2 Differential levels of proteins and lipids between obese individuals and healthy individuals

Some proteins and lipids derived from AT-exos are also different and associated with inflammation in these two groups. As previously stated, adipocyte-exo derived IL-1β, IL-6, IL-8, CCL2, CCL5, TNF-α (3), resistin, and retinol-binding protein 4 (30) are found to be expressed at higher levels in obese individuals. These proteins are considered to be proinflammatory factors and are associated with insulin resistance, and these proteins can also facilitate the M1 polarization of monocytes (30, 40). Furthermore, macrophage migration inhibitory factor, which is expressed at higher levels in the adipocyte-exos from obese individuals (127), is an upstream regulator of the inflammatory cascade and triggers inflammatory responses via migration inhibitory factor signaling pathways (128). Moreover, the plasma levels of adipsin and neuregulin 4 (Nrg4) are decreased in overweight individuals (129, 130). Nrg4 is shown to protect against type 2 diabetes mellitus and non-alcoholic fatty liver disease because Nrg4 can positively regulate ErbB3 and ErbB4 signaling in hepatocytes and inhibit LXR and SREBP1c, promoting lipogenesis (130). In Nrg4-deficient mice with nonalcoholic steatohepatitis, cell death, inflammation, fibrosis, and liver injury are more serious because Nrg4 can positively regulate ErbB3 and ErbB4 to repress the ubiquitination and proteasomal degradation of c-FLIPL to reduce cell death (131). However, the level of palmitoleate, which has certain anti-inflammatory and insulin-sensitizing effects, is downregulated in obese and insulin-resistant mice (132). The proteins and lipids in adipocyte-exos associated with energy metabolism, lipogenesis, and insulin sensitivity are listed in **Table 4**. Moreover, the signal transducer and activator of transcription 3 in ADSC-exos can alleviate AT inflammation and promote the beiging of white AT to improve insulin sensitivity and glucose intolerance during high-fat diet consumption (61).

**Table 4:** Proteins and lipids derived from adipocyte-exos are associated with metabolism and insulin resistance.

Substance		Mechanisms	Reference
Proteins	Adipsin	Complement factor C3a, whose generation depends on adipsin level, can bind with the C3a receptor and activate the Ca^2+^ signaling pathway in pancreatic β cells, thereby accelerating insulin secretion. Obese individuals and T2DM patients exhibit lower circulating adipsin levels, which is thought of as a monitoring factor for patients in the prediabetic state.	(129)
	Mitochondrial FAO enzymes ECHA and HCDH	Adipocytes can provide FAO enzymes, fatty acids, and various autophagic proteins to melanoma cells for energy supply and lipophagy.	(22)
	FABP4	FABP can promote the progression of the obesity-related diseases and combine with AA to form the FABP-AA complex. This complex targets the PPARγ receptor and regulates the target gene transcription in the cell nucleus.	(43, 133)
	FGF21	FGF21 can stimulate the hypothalamic-pituitary-adrenal axis to promote liver gluconeogenesis, ketogenesis, and fatty acid oxidation during fasting. FGF21 in AT-exos can increase heat production and accelerate the development of beige adipocytes under cold stimulation. And the BAT-derived FGF21 has an endocrine regulatory function to reduce cardiac hypertrophy.	(134, 135)
Lipids	FAHFAs	FAHFAs can target G-protein-coupled receptors 40 and 120 to promote insulin secretion and relieve insulin resistance, with decreased levels in obese individuals.	(43, 136)

T2DM, type 2 diabetes mellitus; FAO, fatty acid oxidation; ECHA, trifunctional enzyme subunit alpha; HCDH, hydroxy carboxylic acid dehydrogenase; AA, arachidonic acid; FGF21, fibroblast growth factor 21; BAT, brown adipose tissue; FAHFA, fatty acid ester of hydroxy fatty acid.

**Figure 3:**
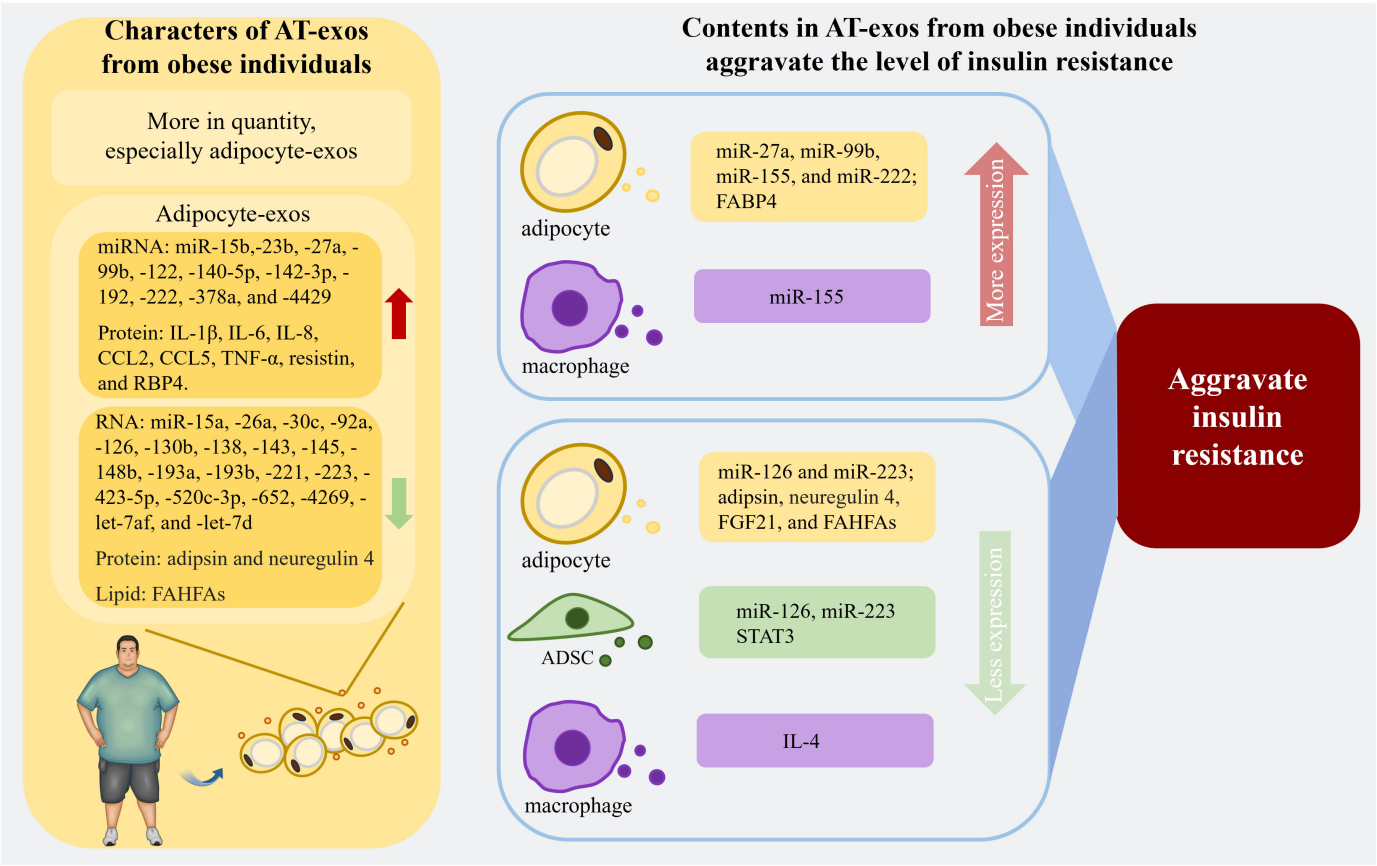
The properties of AT-exos from the obese population and the contents in AT-exos derived from obese individuals are related to the exacerbation of insulin resistance. With an increased quantity, the adipocyte-exos of obese individuals exhibit significant alterations compared to those of normal individuals. The upregulation of certain microRNAs and proteins within these exosomes can exacerbate insulin resistance. Conversely, the downregulation of various RNAs, proteins, and lipid can alleviate insulin resistance, thereby significantly worsening the insulin resistance in obese individuals. Exosomes from ADSCs and macrophages also contain substances that can regulate insulin resistance levels. miR-155 from ATM-exos can inhibit adipogenesis and reduce insulin secretion from pancreatic β cells, aggravating insulin resistance. (Created with BioRender.com). miR-126, miR-223, and STAT3 in ADSC-exos, as well as IL-4 from ATM-exos, are associated with the reduction of inflammation, thereby alleviating insulin resistance. IL-1β, interleukin-1β; IL-6, interleukin-6; IL-8, interleukin-8; CCL2, C-C motif ligand 2; CCL5, C-C motif ligand 5; TNF-α, tumor necrosis factor-α; RBP4, retinol binding protein 4; FAHFAs, fatty acid ester of hydroxy fatty acids; FABP4, fatty acid binding protein 4; FGF21, fibroblast growth factor 21; STAT3, signal transducer and activator of transcription 3; IL-1β, interleukin-1β.

In addition to adipocytes and ADSCs, AT is instrumental in regulating the insulin resistance and glucose sensitivity of individuals. In mouse experiments, injecting ATM-exos from obese mice into lean mice decreased insulin sensitivity and induced glucose tolerance. In contrast, ATM-exos obtained from lean mice improved glucose tolerance and insulin sensitivity when administered to obese mice (25, 121). AT contains multiple types of macrophages that secrete different exosomes according to their unique phenotype. Exosomes from M1 macrophages induce insulin resistance in adipocytes, whereas M2 macrophage-derived exosomes improve insulin sensitivity and glucose tolerance (137). IL-4 derived from THP-1 macrophages can decrease miR-33 expression and upregulate miR-21, miR-99a, miR-146b, and miR-378a in both adipocytes and macrophages to promote lipophagy and oxidative phosphorylation, and these effects are accompanied by enhanced insulin sensitivity (138). High expression of glypican-4 in preadipocytes can result in insulin resistance in the initial stage of obesity (139). Additionally, the increased expression of short stature homeobox 2, which is linked to imbalanced fat storage in subcutaneous adipocytes compared to visceral adipocytes (140), can decrease the expression of the β3 adrenergic receptor, leading to reduce lipolysis (141). According to the findings of these studies, subcutaneous preadipocytes in the obese population can promote more proliferation and accumulation of AT. The cargoes in AT-exos associated with insulin resistance are recapitulated in **Figure 3**.

The modulatory influences of adipocyte-exos, ADSC-exos, and ATM-exos on inflammation, tumor progression, and insulin resistance are concisely detailed in **Table 5**.

**Table 5:** Brief overview of adipocyte-exos, ADSC-exos, and ATM-exos regulating inflammation, tumor progression, and insulin resistance.

Exosomes	Local inflammation	Tumor progression	Insulin resistance
Adipocyte-exos	Aggravation in the obese population	Many compounds can impede tumor progression.	Aggravation in the obese population
ADSC-exos	Alleviation	Some components can deteriorate tumor progression, while others can suppress it.	Alleviation
ATM-exos	Aggravation in the obese population	lncRNA-HISLA can deteriorate tumor progression.	Aggravation in the obese population

lncRNA-HISLA, lncRNA-HIF-1α-stabilizing long noncoding RNA.

## 5 The regulating action of exosomes derived from AT on other tissues and organs

In addition to affecting inflammation, tumors, and insulin resistance, AT can also have regulatory functions on adipocytes themselves, muscle, skin, hypothalamus, and kidney through the delivery of exosomes in the progression of adipogenesis, myodystrophy, skin photoaging, appetite, and renal fibrosis. AT exosomal RNAs play a crucial role in the regulation of adipogenesis (142, 143) and the transformation between white adipocytes and brown adipocytes, such as miR-92a, miR-155, miR-193b, miR-196a, miR-337, and miR-455 (144). Knockout of Dicer, the miRNA-processing enzyme, causes lipodystrophy of visceral and subcutaneous adipocytes, serious insulin resistance, and ‘whitening’ of brown adipocytes in the mouse scapular region (145, 146). Adipocyte-exos could deliver miRNAs to skeletal muscle cells, regulating the metabolism and differentiation of muscle cells. The contents of AT-exos in myodystrophy patients are also different from those in normal individuals (2, 147, 148). Numerous studies have shown that ADSC-exos are beneficial to inflammation-related diseases, which has been confirmed in skin photoaging and renal fibrosis (149, 150). The effective RNAs in AT-exos are listed in **Table 6**.

**Table 6:** AT-exos influence other tissues or organs in diseases or physiological processes.

Tissues/Organs	Disease/Physiological function	RNA	Derived	Mechanisms	Reference
Adipocyte	Adipogenesis	miR-455	Adipocyte and ADSC	miR-455 represses the expression of Necdin, Runx1t1, and the hypoxia-inducible factor 1a inhibitor to activate and promote the brown adipogenic program.	(151)
		lnc-BATE1	Adipocyte	lnc-BATE1 binds heterogeneous nuclear ribonucleoprotein U to promote the differentiation of brown adipocytes.	(152)
	Heat production	miR-92a	Adipocyte	These two miRNAs target and downregulate fibroblast growth factor receptor-1, attenuating the heat-producing capability of BAT.	(153)
		miR-210	ADSC		
Muscles	Myodystrophy	miR-130b	Adipocyte	miR-130b suppresses the expression of peroxisome proliferator-activated receptor-γ coactivator-1α to weaken the lipolysis ability and the oxidative metabolism of skeletal muscle cells.	(154, 155)
		miR-34a-5p, miR-130a-3p, and miR-214-3p	Adipocyte	These miRNAs, muscular dystrophy-associated miRNAs, are expressed more in muscular dystrophy patients and exacerbate tissue fibrosis.	(2, 147, 148)
		miR-125-5p	Adipocyte	miR-125-5p targets insulin-like growth factor 2 and reduces its expression to decelerate muscle differentiation.	(2, 156)
Hypothalamus	Appetite regulation	lncRNA implicated in MALAT1	Adipocyte	MALAT1 activates the mTOR signaling pathway in the hypothalamic pro-opiomelanocortin neurons to increase appetite and energy intake.	(5)
Skin	Skin photoaging	miR-1246	ADSC	miR-1246 targets the MAPK/AP-1 signaling pathway to reduce the production of MMP1 and activates the TGF-β/Smad signaling pathway to enhance the expression of type I collagen.	(149)
Kidney	Diabetic nephropathy	miR-486	ADSC	miR-486 promotes cellular autophagy and alleviates diabetic renal fibrosis by inhibiting the Smad1/mTOR signaling pathway in the podocytes.	(150)

Runx1t1, runt related transcription factor 1 partner transcriptional co-repressor 1; lnc-BATE1, brown adipose tissue enriched long non-coding RNA 1; BAT, brown adipose tissue; MALAT1, metastasis-associated lung adenocarcinoma transcript 1; AP-1, activator protein-1; MMP1, matrix metalloproteinase 1; TGF-β, transforming growth factor β.

Meanwhile, the proteins and lipids in AT-exos also act as regulators in the physiological and pathological processes. Proteins in adipocyte-exos, known as exoadipokines, are associated with inflammation and fibrosis, affect on signaling pathways, and membrane proteins (157). The proteins in both adipocyte-exos and ADSC-exos, as cargoes, can be categorized into regulatory factors and metabolism-related enzymes (110, 158). The enzymes in ADSC-exos can convert adenosine monophosphate to adenosine to activate the PI3K/Akt pathway, exerting a protective effect on myocarditis and arrhythmias (159). Additionally, in tissue infections, ADSCs release the antibacterial peptides and proteins, such as antibacterial peptide LL-37, hepcidin, β-defensin-2, and lipocalin-2, which inhibit the synthesis of DNA, RNA, and valid proteins in infected cells to facilitate the cell-killing process and restore the balance of infection and inflammation (160–162). Lipids in exosomes also play a role in inducing the differentiation of immune cells and regulating gene expression. Lipids in adipocyte-exos promote the differentiation of monocytes from bone marrow into ATM (29). ADSC-exos contain various bioactive lipids, including monounsaturated fatty acids, polyunsaturated fatty acids, and multiple saturated fatty acids. These fatty acids, such as arachidonic acid, prostaglandins, lysophosphatidylcholine, leukotrienes, phosphatidic acid, and docosahexaenoic acid (133), facilitate the transmission of information between cells through different signaling pathways. Arachidonic acid, a common ω-6 polyunsaturated fatty acid, participates in the biosynthesis of prostaglandin. The prostaglandin E2-EP3, found in bone marrow mesenchymal stem cells with selective secretion (163), can induce acute inflammation performance (164). Furthermore, prostaglandin E2 can also accelerate tumor cell proliferation (165). In clinical trials, high expression of docosahexaenoic acid is related to better chemotherapy effect in BC and non-small-cell lung cancer patients (166).

## 6 Clinical implications of AT-exos

Exosomes, which are characterized by low immunogenicity, regulate recipient cells by delivering cargoes to achieve cell-free therapy. Both utilizing the substances contained in exosomes and delivering drugs through exosomes are beneficial methods for disease treatment. AT-exos were proven to be therapeutic for wound healing by promoting the proliferation and migration of fibroblasts and HaCaT cells as well as angiogenesis (19). AT-exo-derived lnc-H19 (167) and miR-221-3p (27) can promote cell proliferation and vascularization, respectively. Furthermore, AT-exos have potential therapeutic benefits for liver injury, cardiac fibrosis, metabolic syndrome, and tumors (168, 169). As mentioned above, AT-exos are present at different levels in obese and diabetic people than in healthy people and could serve as a basis for identifying potentially diabetic patients from healthy or obese people.

However, there are several limitations in the clinical application of AT-exos. First, the stability of AT-exos during storage is challenging because exosomes are susceptible to environmental conditions during storage and degrade over time, which limits their long-term storage and wide application. Moreover, there is no consensus about preparation methods and quality control standards of AT-exos, which leads to uncertainty about the quality and efficacy of exosomes, limiting their reliability and reproducibility in clinical application. Finally, although AT-exos are considered relatively safe, they have potential safety concerns and side effects, such as immune responses or other adverse reactions. The safety, dosage, and treatment options of AT-exos need to be further studied and evaluated in additional animal and clinical experiments.

Compared with AT-exos, ADSC-exos have been extensively studied in clinical applications, and they have strong and wide-ranging impacts. In addition to promoting cell proliferation and migration as well as angiogenesis in wound healing (170), ADSC-exos have great curative effects on inflammation-related diseases (Crohn’s disease, idiopathic pulmonary fibrosis, COVID-19, arthritis, autoimmune diabetes, etc.), myocardial ischemia, and delayed photoaging (3, 150, 160, 171). Unlike ADSC-exos, some studies have shown that AT-exos in obese people could increase inflammation and exacerbate disease (172, 173). So, the efficacy of AT-exos from obese people in the treatment of inflammatory diseases is still controversial. Ultimately, compared with ADSC-exos, AT-exos have great advantages in terms of preparation. AT-exos are directly extracted from AT without the need for cell expansion, meaning that the time required for AT-exo preparation is significantly shorter than that required for ADSC-exo preparation. AT-exos, which are exosomes in mature tissue, are significantly more abundant than ADSC-exos produced by primary ADSCs that are extracted from the same volume of AT.

## 7 Conclusions and prospects

AT-exos, which are produced by various AT cells, mediate paracrine and endocrine regulation of the local microenvironment and distant organs by delivering nucleic acids, proteins, and lipids. Among AT-exos, adipocyte-exos and ADSC-exos have positive impacts on wound healing and cardiovascular protection, while their regulatory effects on inflammation and tumors are complicated. Furthermore, the contents of AT-exos significantly differ among healthy individuals, obese individuals, and diabetic patients. These differences could serve as targets for identifying patients with early-stage diabetes. Modulating the expression of insulin-related genes may improve the insulin sensitivity of patients and contribute to the treatment of diabetes and obesity.

Various cells within AT generate exosomes that perform diverse regulatory functions. Considering AT-exos as a whole is valuable for studying the regulatory effect of AT on other organs in the body. In order to better understand the regulatory role of AT-exos and take advantage of AT-exos, further studies could focus on the following directions: (1) Identifying appropriate patients with inflammation-related diseases for treatment with autologous AT-exos due to their complex regulatory effects of AT-exos on inflammation. (2) Investigating whether AT-exos exacerbate tumor progression and increase the risk of recurrence in BC patients with fat breast augmentation after resection. (3) contents, such as miRNAs, proteins, and lipids, in AT-exos can be used to predict whether obese patients will suffer from diabetes, although the expression of miRNAs is easily affected by stimulation. (4) Enhancing the separation and purification techniques of exosomes from various cellular sources to facilitate the development of targeted therapies and achieve desired treatment outcomes. (5) Improving methods for the preparation and storage of AT-exos to ensure uniformity, which will greatly enhance the clinical application of AT-exos. Addressing these key questions will pave the way for the development and more efficient use of autologous AT-exos for treating diseases in clinical practice.

## References

[1] Kruglikov IL Zhang Z Scherer PE. The role of immature and mature adipocytes in hair cycling. Trends Endocrinol Metabol: TEM. (2019) 30:93–105. doi: 10.1016/j.tem.2018.11.004 PMC6348020 30558832

[2] Ojima K Muroya S Wada H Ogawa K Oe M Takimoto K. Immature adipocyte-derived exosomes inhibit expression of muscle differentiation markers. FEBS Open Bio. (2021) 11:768–81. doi: 10.1002/2211-5463.13100 PMC7931241 33527775

[3] Huang Z Xu A. Adipose extracellular vesicles in intercellular and inter-organ crosstalk in metabolic health and diseases. Front Immunol. (2021) 12:608680. doi: 10.3389/fimmu.2021.608680 33717092 PMC7946830

[4] Pan Y Hui X Hoo RLC Ye D Chan CYC Feng T. Adipocyte-secreted exosomal microRNA-34a inhibits M2 macrophage polarization to promote obesity-induced adipose inflammation. J Clin Invest. (2019) 129:834–49. doi: 10.1172/jci123069 PMC6355214 30667374

[5] Gao J Li X Wang Y Cao Y Yao D Sun L. Adipocyte-derived extracellular vesicles modulate appetite and weight through mTOR signalling in the hypothalamus. Acta physiologica (Oxford England). (2020) 228:e13339. doi: 10.1111/apha.13339 31278836

[6] Gesmundo I Pardini B Gargantini E Gamba G Birolo G Fanciulli A. Adipocyte-derived extracellular vesicles regulate survival and function of pancreatic β cells. JCI Insight. (2021) 6:e141962. doi: 10.1172/jci.insight.141962 33539327 PMC8021102

[7] Li Y He X Li Q Lai H Zhang H Hu Z. EV-origin: Enumerating the tissue-cellular origin of circulating extracellular vesicles using exLR profile. Comput Struct Biotechnol J. (2020) 18:2851–9. doi: 10.1016/j.csbj.2020.10.002 PMC7588739 33133426

[8] Connolly KD Wadey RM Mathew D Johnson E Rees DA James PE. Evidence for adipocyte-derived extracellular vesicles in the human circulation. Endocrinology. (2018) 159:3259–67. doi: 10.1210/en.2018-00266 PMC6109300 30016424

[9] Jeppesen DK Zhang Q Franklin JL Coffey RJ. Extracellular vesicles and nanoparticles: emerging complexities. Trends Cell Biol. (2023) 33:667–81. doi: 10.1016/j.tcb.2023.01.002 PMC10363204 36737375

[10] Théry C Witwer KW Aikawa E Alcaraz MJ Anderson JD Andriantsitohaina R. Minimal information for studies of extracellular vesicles 2018 (MISEV2018): a position statement of the International Society for Extracellular Vesicles and update of the MISEV2014 guidelines. J Extracell Vesic. (2018) 7:1535750. doi: 10.1080/20013078.2018.1535750 PMC6322352 30637094

[11] Verweij FJ Bebelman MP George AE Couty M Bécot A Palmulli R. ER membrane contact sites support endosomal small GTPase conversion for exosome secretion. J Cell Biol. (2022) 221:e202112032. doi: 10.1083/jcb.202112032 36136097 PMC9507465

[12] Yu W Hurley J Roberts D Chakrabortty SK Enderle D Noerholm M. Exosome-based liquid biopsies in cancer: opportunities and challenges. Ann Oncol: Off J Eur Soc Med Oncol. (2021) 32:466–77. doi: 10.1016/j.annonc.2021.01.074 PMC8268076 33548389

[13] Crewe C Joffin N Rutkowski JM Kim M Zhang F Towler DA. An endothelial-to-adipocyte extracellular vesicle axis governed by metabolic state. Cell. (2018) 175:695–708.e13. doi: 10.1016/j.cell.2018.09.005 30293865 PMC6195477

[14] Al-Ghadban S Bunnell BA. Adipose tissue-derived stem cells: immunomodulatory effects and therapeutic potential. Physiol (Bethesda Md). (2020) 35:125–33. doi: 10.1152/physiol.00021.2019 32027561

[15] Kawada-Horitani E Kita S Okita T Nakamura Y Nishida H Honma Y. Human adipose-derived mesenchymal stem cells prevent type 1 diabetes induced by immune checkpoint blockade. Diabetologia. (2022) 65:1185–97. doi: 10.1007/s00125-022-05708-3 PMC9174328 35511238

[16] Alió Del Barrio JL de la Mata A De Miguel MP Arnalich-Montiel F Nieto-Miguel T El Zarif M. Corneal regeneration using adipose-derived mesenchymal stem cells. Cells. (2022) 11:2549. doi: 10.3390/cells11162549 36010626 PMC9406486

[17] Sanz-Ros J Romero-García N Mas-Bargues C Monleón D Gordevicius J Brooke RT. Small extracellular vesicles from young adipose-derived stem cells prevent frailty, improve health span, and decrease epigenetic age in old mice. Sci Adv. (2022) 8:eabq2226. doi: 10.1126/sciadv.abq2226 36260670 PMC9581480

[18] Wang J Wu H Peng Y Zhao Y Qin Y Zhang Y. Hypoxia adipose stem cell-derived exosomes promote high-quality healing of diabetic wound involves activation of PI3K/Akt pathways. J Nanobiotechnol. (2021) 19:202. doi: 10.1186/s12951-021-00942-0 PMC8261989 34233694

[19] Pan C Xu P Zheng Y Wang Y Chen C Fu S. Preparation of therapy-grade extracellular vesicles from adipose tissue to promote diabetic wound healing. Front Bioeng Biotechnol. (2023) 11:1129187. doi: 10.3389/fbioe.2023.1129187 37034267 PMC10076785

[20] Liu Y Tan J Ou S Chen J Chen L. Adipose-der ived exosomes deliver miR-23a/b to regulate tumor growth in hepatocellular cancer by targeting the VHL/HIF axis. J Physiol Biochem. (2019) 75:391–401. doi: 10.1007/s13105-019-00692-6 31321740

[21] Lin Z Wu Y Xu Y Li G Li Z Liu T. Mesenchymal stem cell-derived exosomes in cancer therapy resistance: recent advances and therapeutic potential. Mol Cancer. (2022) 21:179. doi: 10.1186/s12943-022-01650-5 36100944 PMC9468526

[22] Clement E Lazar I Attané C Carrié L Dauvillier S Ducoux-Petit M. Adipocyte extracellular vesicles carry enzymes and fatty acids that stimulate mitochondrial metabolism and remodeling in tumor cells. EMBO J. (2020) 39:e102525. doi: 10.15252/embj.2019102525 31919869 PMC6996584

[23] Lai RC Chen TS Lim SK. Mesenchymal stem cell exosome: a novel stem cell-based therapy for cardiovascular disease. Regenerative Med. (2011) 6:481–92. doi: 10.2217/rme.11.35 21749206

[24] Alcedo KP Rouse MA Jung GS Fu D Minor M Willcockson HH. CD73 maintains hepatocyte metabolic integrity and mouse liver homeostasis in a sex-dependent manner. Cell Mol Gastroenterol Hepatol. (2021) 12:141–57. doi: 10.1016/j.jcmgh.2021.01.016 PMC8082562 33516905

[25] Liu T Sun YC Cheng P Shao HG. Adipose tissue macrophage-derived exosomal miR-29a regulates obesity-associated insulin resistance. Biochem Biophys Res Commun. (2019) 515:352–8. doi: 10.1016/j.bbrc.2019.05.113 31153636

[26] Xourafa G Korbmacher M Roden M. Inter-organ crosstalk during development and progression of type 2 diabetes mellitus. Nat Rev Endocrinol. (2024) 20:27–49. doi: 10.1038/s41574-023-00898-1 37845351

[27] Li X Ballantyne LL Yu Y Funk CD. Perivascular adipose tissue-derived extracellular vesicle miR-221-3p mediates vascular remodeling. FASEB journal: Off Publ Fed Am Societies Exp Biol. (2019) 33:12704–22. doi: 10.1096/fj.201901548R PMC6902668 31469602

[28] Fuchs A Samovski D Smith GI Cifarelli V Farabi SS Yoshino J. Associations among adipose tissue immunology, inflammation, exosomes and insulin sensitivity in people with obesity and nonalcoholic fatty liver disease. Gastroenterology. (2021) 161:968–81.e12. doi: 10.1053/j.gastro.2021.05.008 34004161 PMC8900214

[29] Flaherty SE 3rd Grijalva A Xu X Ables E Nomani A Ferrante AW Jr. A lipase-independent pathway of lipid release and immune modulation by adipocytes. Sci (New York NY). (2019) 363:989–93. doi: 10.1126/science.aaw2586 PMC6579605 30819964

[30] Deng ZB Poliakov A Hardy RW Clements R Liu C Liu Y. Adipose tissue exosome-like vesicles mediate activation of macrophage-induced insulin resistance. Diabetes. (2009) 58:2498–505. doi: 10.2337/db09-0216 PMC2768161 19675137

[31] Castaño C Kalko S Novials A Párrizas M. Obesity-associated exosomal miRNAs modulate glucose and lipid metabolism in mice. Proc Natl Acad Sci USA. (2018) 115:12158–63. doi: 10.1073/pnas.1808855115 PMC6275521 30429322

[32] Togliatto G Dentelli P Gili M Gallo S Deregibus C Biglieri E. Obesity reduces the pro-angiogenic potential of adipose tissue stem cell-derived extracellular vesicles (EVs) by impairing miR-126 content: impact on clinical applications. Int J Obes. (2016) 40:102–11. doi: 10.1038/ijo.2015.123 PMC4722244 26122028

[33] Kulyté A Belarbi Y Lorente-Cebrián S Bambace C Arner E Daub CO. Additive effects of microRNAs and transcription factors on CCL2 production in human white adipose tissue. Diabetes. (2014) 63:1248–58. doi: 10.2337/db13-0702 24379347

[34] Arner E Mejhert N Kulyté A Balwierz PJ Pachkov M Cormont M. Adipose tissue microRNAs as regulators of CCL2 production in human obesity. Diabetes. (2012) 61:1986–93. doi: 10.2337/db11-1508 PMC3402332 22688341

[35] Huang JH Fu CH Xu Y Yin XM Cao Y Lin FY. Extracellular vesicles derived from epidural fat-mesenchymal stem cells attenuate NLRP3 inflammasome activation and improve functional recovery after spinal cord injury. Neurochem Res. (2020) 45:760–71. doi: 10.1007/s11064-019-02950-x 31953741

[36] Yu C Chen P Xu J Liu Y Li H Wang L. hADSCs derived extracellular vesicles inhibit NLRP3inflammasome activation and dry eye. Sci Rep. (2020) 10:14521. doi: 10.1038/s41598-020-71337-8 32884023 PMC7471690

[37] Chen B Cai J Wei Y Jiang Z Desjardins HE Adams AE. Exosomes are comparable to source adipose stem cells in fat graft retention with up-regulating early inflammation and angiogenesis. Plast Reconstruct Surg. (2019) 144:816e–27e. doi: 10.1097/prs.0000000000006175 31385891

[38] Zhou X Zhang J Lv W Zhao C Xia Y Wu Y. The pleiotropic roles of adipocyte secretome in remodeling breast cancer. J Exp Clin Cancer Res: CR. (2022) 41:203. doi: 10.1186/s13046-022-02408-z 35701840 PMC9199207

[39] Wu Q Li B Li Z Li J Sun S Sun S. Cancer-associated adipocytes: key players in breast cancer progression. J Hematol Oncol. (2019) 12:95. doi: 10.1186/s13045-019-0778-6 31500658 PMC6734503

[40] Blaszczak AM Jalilvand A Hsueh WA. Adipocytes, innate immunity and obesity: A mini-review. Front Immunol. (2021) 12:650768. doi: 10.3389/fimmu.2021.650768 34248937 PMC8264354

[41] Trzyna A Banaś-Ząbczyk A. Adipose-derived stem cells secretome and its potential application in “Stem cell-free therapy. Biomolecules. (2021) 11:878. doi: 10.3390/biom11060878 34199330 PMC8231996

[42] Blaber SP Webster RA Hill CJ Breen EJ Kuah D Vesey G. Analysis of in *vitro* secretion profiles from adipose-derived cell populations. J Trans Med. (2012) 10:172. doi: 10.1186/1479-5876-10-172 PMC3479070 22913454

[43] Scheja L Heeren J. The endocrine function of adipose tissues in health and cardiometabolic disease. Nat Rev Endocrinol. (2019) 15:507–24. doi: 10.1038/s41574-019-0230-6 31296970

[44] Jussila A Zhang B Kirti S Atit R. Tissue fibrosis associated depletion of lipid-filled cells. Exp Dermatol. (2024) 33:e15054. doi: 10.1111/exd.15054 38519432 PMC10977660

[45] Chang X Wang L Wang Z Wu S Zhu X Hu S. TRADD mediates the tumor necrosis factor-induced apoptosis of L929 cells in the absence of RIP3. Sci Rep. (2017) 7:16111. doi: 10.1038/s41598-017-16390-6 29170425 PMC5701027

[46] Song Y Li H Ren X Li H Feng C. SNHG9, delivered by adipocyte-derived exosomes, alleviates inflammation and apoptosis of endothelial cells through suppressing TRADD expression. Eur J Pharmacol. (2020) 872:172977. doi: 10.1016/j.ejphar.2020.172977 32007500

[47] Hu J Jiang Y Wu X Wu Z Qin J Zhao Z. Exosomal miR-17-5p from adipose-derived mesenchymal stem cells inhibits abdominal aortic aneurysm by suppressing TXNIP-NLRP3 inflammasome. Stem Cell Res Ther. (2022) 13:349. doi: 10.1186/s13287-022-03037-1 35883151 PMC9327292

[48] Liu Y Zhang Z Wang B Dong Y Zhao C Zhao Y. Inflammation-stimulated MSC-derived small extracellular vesicle miR-27b-3p regulates macrophages by targeting CSF-1 to promote temporomandibular joint condylar regeneration. Small (Weinheim an der Bergstrasse Germany). (2022) 18:e2107354. doi: 10.1002/smll.202107354 35277920

[49] Li R Li D Wang H Chen K Wang S Xu J. Exosomes from adipose-derived stem cells regulate M1/M2 macrophage phenotypic polarization to promote bone healing via miR-451a/MIF. Stem Cell Res Ther. (2022) 13:149. doi: 10.1186/s13287-022-02823-1 35395782 PMC8994256

[50] Gao Y Mi N Zhang Y Li X Guan W Bai C. Uterine macrophages as treatment targets for therapy of premature rupture of membranes by modified ADSC-EVs through a circRNA/miRNA/NF-κB pathway. J Nanobiotechnol. (2022) 20:487. doi: 10.1186/s12951-022-01696-z PMC9675163 36402996

[51] Zhuang G Meng C Guo X Cheruku PS Shi L Xu H. A novel regulator of macrophage activation: miR-223 in obesity-associated adipose tissue inflammation. Circulation. (2012) 125:2892–903. doi: 10.1161/circulationaha.111.087817 22580331

[52] Niu Q Wang T Wang Z Wang F Huang D Sun H. Adipose-derived mesenchymal stem cell-secreted extracellular vesicles alleviate non-alcoholic fatty liver disease via delivering miR-223-3p. Adipocyte. (2022) 11:572–87. doi: 10.1080/21623945.2022.2098583 PMC9481107 36093813

[53] Liu W Liu A Li X Sun Z Sun Z Liu Y. Dual-engineered cartilage-targeting extracellular vesicles derived from mesenchymal stem cells enhance osteoarthritis treatment via miR-223/NLRP3/pyroptosis axis: Toward a precision therapy. Bioactive Mater. (2023) 30:169–83. doi: 10.1016/j.bioactmat.2023.06.012 PMC10429745 37593145

[54] Zhang Y Mei H Chang X Chen F Zhu Y Han X. Adipocyte-derived microvesicles from obese mice induce M1 macrophage phenotype through secreted miR-155. J Mol Cell Biol. (2016) 8:505–17. doi: 10.1093/jmcb/mjw040 27671445

[55] Zhao YQ Ren YF Li BB Wei C Yu B. The mysterious association between adiponectin and endometriosis. Front Pharmacol. (2024) 15:1396616. doi: 10.3389/fphar.2024.1396616 38813109 PMC11133721

[56] Chedid P Hurtado-Nedelec M Marion-Gaber B Bournier O Hayem G Gougerot-Pocidalo MA. Adiponectin and its globular fragment differentially modulate the oxidative burst of primary human phagocytes. Am J pathology. (2012) 180:682–92. doi: 10.1016/j.ajpath.2011.10.013 22119038

[57] Trellakis S Rydleuskaya A Fischer C Canbay A Tagay S Scherag A. Low adiponectin, high levels of apoptosis and increased peripheral blood neutrophil activity in healthy obese subjects. Obes facts. (2012) 5:305–18. doi: 10.1159/000339452 22722748

[58] Duan Y Zhang S Xing Y Wu Y Zhao W Xie P. Adiponectin-mediated promotion of CD44 suppresses diabetic vascular inflammatory effects. iScience. (2023) 26:106428. doi: 10.1016/j.isci.2023.106428 37020952 PMC10067764

[59] Qiu W Wu H Hu Z Wu X Tu M Fang F. Identification and characterization of a novel adiponectin receptor agonist adipo anti-inflammation agonist and its anti-inflammatory effects in *vitro* and in vivo. Br J Pharmacol. (2021) 178:280–97. doi: 10.1111/bph.15277 PMC8364772 32986862

[60] Liu Z Gan L Zhang T Ren Q Sun C. Melatonin alleviates adipose inflammation through elevating α-ketoglutarate and diverting adipose-derived exosomes to macrophages in mice. J pineal Res. (2018) 64:e12455. doi: 10.1111/jpi.12455 29149454

[61] Zhao H Shang Q Pan Z Bai Y Li Z Zhang H. Exosomes from adipose-derived stem cells attenuate adipose inflammation and obesity through polarizing M2 macrophages and beiging in white adipose tissue. Diabetes. (2018) 67:235–47. doi: 10.2337/db17-0356 29133512

[62] Endo J Sano M Isobe Y Fukuda K Kang JX Arai H. 18-HEPE, an n-3 fatty acid metabolite released by macrophages, prevents pressure overload-induced maladaptive cardiac remodeling. J Exp Med. (2014) 211:1673–87. doi: 10.1084/jem.20132011 PMC4113943 25049337

[63] Dayakar A Chandrasekaran S Veronica J Maurya R. Leptin induces the phagocytosis and protective immune response in Leishmania donovani infected THP-1 cell line and human PBMCs. Exp Parasitol. (2016) 160:54–9. doi: 10.1016/j.exppara.2015.12.002 26688099

[64] Ninou I Magkrioti C Aidinis V. Autotaxin in pathophysiology and pulmonary fibrosis. Front Med. (2018) 5:180. doi: 10.3389/fmed.2018.00180 PMC6008954 29951481

[65] Ma H Li YN Song L Liu R Li X Shang Q. Macrophages inhibit adipogenic differentiation of adipose tissue derived mesenchymal stem/stromal cells by producing pro-inflammatory cytokines. Cell Biosci. (2020) 10:88. doi: 10.1186/s13578-020-00450-y 32699606 PMC7372775

[66] Su J Chen X Huang Y Li W Li J Cao K. Phylogenetic distinction of iNOS and IDO function in mesenchymal stem cell-mediated immunosuppression in mammalian species. Cell Death Differ. (2014) 21:388–96. doi: 10.1038/cdd.2013.149 PMC3921585 24162664

[67] Park HK Kwak MK Kim HJ Ahima RS. Linking resistin, inflammation, and cardiometabolic diseases. Korean J Internal Med. (2017) 32:239–47. doi: 10.3904/kjim.2016.229 PMC5339472 28192887

[68] Schwartz DR Lazar MA. Human resistin: found in translation from mouse to man. Trends Endocrinol Metabolism: TEM. (2011) 22:259–65. doi: 10.1016/j.tem.2011.03.005 PMC3130099 21497511

[69] Benomar Y Amine H Crépin D Al Rifai S Riffault L Gertler A. Central resistin/TLR4 impairs adiponectin signaling, contributing to insulin and FGF21 resistance. Diabetes. (2016) 65:913–26. doi: 10.2337/db15-1029 26740596

[70] Wang S Su X Xu M Xiao X Li X Li H. Exosomes secreted by mesenchymal stromal/stem cell-derived adipocytes promote breast cancer cell growth via activation of Hippo signaling pathway. Stem Cell Res Ther. (2019) 10:117. doi: 10.1186/s13287-019-1220-2 30971292 PMC6458638

[71] Huang R Wang Z Hong J Wu J Huang O He J. Targeting cancer-associated adipocyte-derived CXCL8 inhibits triple-negative breast cancer progression and enhances the efficacy of anti-PD-1 immunotherapy. Cell Death Dis. (2023) 14:703. doi: 10.1038/s41419-023-06230-z 37898619 PMC10613226

[72] Tucker SL Gharpure K Herbrich SM Unruh AK Nick AM Crane EK. Molecular biomarkers of residual disease after surgical debulking of high-grade serous ovarian cancer. Clin Cancer research: an Off J Am Assoc Cancer Res. (2014) 20:3280–8. doi: 10.1158/1078-0432.ccr-14-0445 PMC4062703 24756370

[73] García-Contreras M Vera-Donoso CD Hernández-Andreu JM García-Verdugo JM Oltra E. Therapeutic potential of human adipose-derived stem cells (ADSCs) from cancer patients: a pilot study. PloS One. (2014) 9:e113288. doi: 10.1371/journal.pone.0113288 25412325 PMC4239050

[74] Lin R Wang S Zhao RC. Exosomes from human adipose-derived mesenchymal stem cells promote migration through Wnt signaling pathway in a breast cancer cell model. Mol Cell Biochem. (2013) 383:13–20. doi: 10.1007/s11010-013-1746-z 23812844

[75] García Garre E Luengo Gil G Montoro García S Gonzalez Billalabeitia E Zafra Poves M García Martinez E. Circulating small-sized endothelial microparticles as predictors of clinical outcome after chemotherapy for breast cancer: an exploratory analysis. Breast Cancer Res Treat. (2018) 169:83–92. doi: 10.1007/s10549-017-4656-z 29340882

[76] Ko SF Yip HK Zhen YY Lee CC Lee CC Huang CC. Adipose-derived mesenchymal stem cell exosomes suppress hepatocellular carcinoma growth in a rat model: apparent diffusion coefficient, natural killer T-cell responses, and histopathological features. Stem Cells Int. (2015) 2015:853506. doi: 10.1155/2015/853506 26345219 PMC4545422

[77] Yoshida K Yokoi A Kato T Ochiya T Yamamoto Y. The clinical impact of intra- and extracellular miRNAs in ovarian cancer. Cancer Sci. (2020) 111:3435–44. doi: 10.1111/cas.14599 PMC7541008 32750177

[78] Heyn GS Corrêa LH Magalhães KG. The impact of adipose tissue-derived miRNAs in metabolic syndrome, obesity, and cancer. Front Endocrinol. (2020) 11:563816. doi: 10.3389/fendo.2020.563816 PMC7573351 33123088

[79] Ono M Kosaka N Tominaga N Yoshioka Y Takeshita F Takahashi RU. Exosomes from bone marrow mesenchymal stem cells contain a microRNA that promotes dormancy in metastatic breast cancer cells. Sci Signal. (2014) 7:ra63. doi: 10.1126/scisignal.2005231 24985346

[80] Phan TG Croucher PI. The dormant cancer cell life cycle. Nat Rev Cancer. (2020) 20:398–411. doi: 10.1038/s41568-020-0263-0 32488200

[81] Li Y Zhao Z Liu W Li X. SNHG3 functions as miRNA sponge to promote breast cancer cells growth through the metabolic reprogramming. Appl Biochem Biotechnol. (2020) 191:1084–99. doi: 10.1007/s12010-020-03244-7 PMC7320061 31956955

[82] Yang E Wang X Gong Z Yu M Wu H Zhang D. Exosome-mediated metabolic reprogramming: the emerging role in tumor microenvironment remodeling and its influence on cancer progression. Signal Transduct Target Ther. (2020) 5:242. doi: 10.1038/s41392-020-00359-5 33077737 PMC7572387

[83] Chen F Chen J Yang L Liu J Zhang X Zhang Y. Extracellular vesicle-packaged HIF-1α-stabilizing lncRNA from tumour-associated macrophages regulates aerobic glycolysis of breast cancer cells. Nat Cell Biol. (2019) 21:498–510. doi: 10.1038/s41556-019-0299-0 30936474

[84] Wang Z He J Bach DH Huang YH Li Z Liu H. Induction of m(6)A methylation in adipocyte exosomal LncRNAs mediates myeloma drug resistance. J Exp Clin Cancer Res: CR. (2022) 41:4. doi: 10.1186/s13046-021-02209-w 34980213 PMC8722039

[85] Guaita-Esteruelas S Gumà J Masana L Borràs J. The peritumoural adipose tissue microenvironment and cancer. The roles of fatty acid binding protein 4 and fatty acid binding protein 5. Mol Cell Endocrinol. (2018) 462:107–18. doi: 10.1016/j.mce.2017.02.002 28163102

[86] Nieman KM Kenny HA Penicka CV Ladanyi A Buell-Gutbrod R Zillhardt MR. Adipocytes promote ovarian cancer metastasis and provide energy for rapid tumor growth. Nat Med. (2011) 17:1498–503. doi: 10.1038/nm.2492 PMC4157349 22037646

[87] Afrin S Ramaiyer M Begum UAM Borahay MA. Adipocyte and adipokines promote a uterine leiomyoma friendly microenvironment. Nutrients. (2023) 15:715. doi: 10.3390/nu15030715 36771423 PMC9919329

[88] Jafari N Kolla M Meshulam T Shafran JS Qiu Y Casey AN. Adipocyte-derived exosomes may promote breast cancer progression in type 2 diabetes. Sci Signaling. (2021) 14:eabj2807. doi: 10.1126/scisignal.abj2807 PMC8765301 34813359

[89] Wang L Tang C Cao H Li K Pang X Zhong L. Activation of IL-8 via PI3K/Akt-dependent pathway is involved in leptin-mediated epithelial-mesenchymal transition in human breast cancer cells. Cancer Biol Ther. (2015) 16:1220–30. doi: 10.1080/15384047.2015.1056409 PMC4622725 26121010

[90] He JY Wei XH Li SJ Liu Y Hu HL Li ZZ. Adipocyte-derived IL-6 and leptin promote breast Cancer metastasis via upregulation of Lysyl Hydroxylase-2 expression. Cell Commun Signal.: CCS. (2018) 16:100. doi: 10.1186/s12964-018-0309-z 30563531 PMC6299564

[91] Chen C Chang YC Lan MS Breslin M. Leptin stimulates ovarian cancer cell growth and inhibits apoptosis by increasing cyclin D1 and Mcl-1 expression via the activation of the MEK/ERK1/2 and PI3K/Akt signaling pathways. Int J Oncol. (2013) 42:1113–9. doi: 10.3892/ijo.2013.1789 23354006

[92] Montagner A Polizzi A Fouché E Ducheix S Lippi Y Lasserre F. Liver PPARα is crucial for whole-body fatty acid homeostasis and is protective against NAFLD. Gut. (2016) 65:1202–14. doi: 10.1136/gutjnl-2015-310798 PMC4941147 26838599

[93] Vallabhaneni KC Penfornis P Dhule S Guillonneau F Adams KV Mo YY. Extracellular vesicles from bone marrow mesenchymal stem/stromal cells transport tumor regulatory microRNA, proteins, and metabolites. Oncotarget. (2015) 6:4953–67. doi: 10.18632/oncotarget.3211 PMC4467126 25669974

[94] Wan FZ Chen KH Sun YC Chen XC Liang RB Chen L. Exosomes overexpressing miR-34c inhibit Malignant behavior and reverse the radioresistance of nasopharyngeal carcinoma. J Trans Med. (2020) 18:12. doi: 10.1186/s12967-019-02203-z PMC6947927 31915008

[95] Lou G Song X Yang F Wu S Wang J Chen Z. Exosomes derived from miR-122-modified adipose tissue-derived MSCs increase chemosensitivity of hepatocellular carcinoma. J Hematol Oncol. (2015) 8:122. doi: 10.1186/s13045-015-0220-7 26514126 PMC4627430

[96] Lee HK Finniss S Cazacu S Bucris E Ziv-Av A Xiang C. Mesenchymal stem cells deliver synthetic microRNA mimics to glioma cells and glioma stem cells and inhibit their cell migration and self-renewal. Oncotarget. (2013) 4:346–61. doi: 10.18632/oncotarget.868 PMC3712579 23548312

[97] Qian C Wang Y Ji Y Chen D Wang C Zhang G. Neural stem cell−derived exosomes transfer miR−124−3p into cells to inhibit glioma growth by targeting FLOT2. Int J Oncol. (2022) 61:115. doi: 10.3892/ijo.2022.5405 35929514 PMC9387557

[98] Takahara K Ii M Inamoto T Nakagawa T Ibuki N Yoshikawa Y. microRNA-145 mediates the inhibitory effect of adipose tissue-derived stromal cells on prostate cancer. Stem Cells Dev. (2016) 25:1290–8. doi: 10.1089/scd.2016.0093 27465939

[99] Lou G Chen L Xia C Wang W Qi J Li A. MiR-199a-modified exosomes from adipose tissue-derived mesenchymal stem cells improve hepatocellular carcinoma chemosensitivity through mTOR pathway. J Exp Clin Cancer research: CR. (2020) 39:4. doi: 10.1186/s13046-019-1512-5 31898515 PMC6941283

[100] Yu L Gui S Liu Y Qiu X Zhang G Zhang X. Exosomes derived from microRNA-199a-overexpressing mesenchymal stem cells inhibit glioma progression by down-regulating AGAP2. Aging. (2019) 11:5300–18. doi: 10.18632/aging.102092 PMC6710058 31386624

[101] Seo M Kim SM Woo EY Han KC Park EJ Ko S. Stemness-attenuating miR-503-3p as a paracrine factor to regulate growth of cancer stem cells. Stem Cells Int. (2018) 2018:4851949. doi: 10.1155/2018/4851949 29849663 PMC5904772

[102] Jia Z Zhu H Sun H Hua Y Zhang G Jiang J. Adipose mesenchymal stem cell-derived exosomal microRNA-1236 reduces resistance of breast cancer cells to cisplatin by suppressing SLC9A1 and the wnt/β-catenin signaling. Cancer Manage Res. (2020) 12:8733–44. doi: 10.2147/cmar.s270200 PMC7519869 33061571

[103] Kranendonk ME Visseren FL van Balkom BW Nolte-’t Hoen EN van Herwaarden JA de Jager W. Human adipocyte extracellular vesicles in reciprocal signaling between adipocytes and macrophages. Obes (Silver Spring Md). (2014) 22:1296–308. doi: 10.1002/oby.20679 24339422

[104] Trajkovic K Hsu C Chiantia S Rajendran L Wenzel D Wieland F. Ceramide triggers budding of exosome vesicles into multivesicular endosomes. Sci (New York NY). (2008) 319:1244–7. doi: 10.1126/science.1153124 18309083

[105] Egea-Jimenez AL Zimmermann P. Phospholipase D and phosphatidic acid in the biogenesis and cargo loading of extracellular vesicles. J Lipid Res. (2018) 59:1554–60. doi: 10.1194/jlr.R083964 PMC6121939 29853529

[106] Li Y Talbot CL Chaurasia B. Ceramides in adipose tissue. Front Endocrinol. (2020) 11:407. doi: 10.3389/fendo.2020.00407 PMC7316884 32636806

[107] Kim JI Huh JY Sohn JH Choe SS Lee YS Lim CY. Lipid-overloaded enlarged adipocytes provoke insulin resistance independent of inflammation. Mol Cell Biol. (2015) 35:1686–99. doi: 10.1128/mcb.01321-14 PMC4405637 25733684

[108] Lazar I Clement E Dauvillier S Milhas D Ducoux-Petit M LeGonidec S. Adipocyte exosomes promote melanoma aggressiveness through fatty acid oxidation: A novel mechanism linking obesity and cancer. Cancer Res. (2016) 76:4051–7. doi: 10.1158/0008-5472.can-16-0651 27216185

[109] Durcin M Fleury A Taillebois E Hilairet G Krupova Z Henry C. Characterisation of adipocyte-derived extracellular vesicle subtypes identifies distinct protein and lipid signatures for large and small extracellular vesicles. J Extracell Vesic. (2017) 6:1305677. doi: 10.1080/20013078.2017.1305677 PMC5405565 28473884

[110] Kwan HY Chen M Xu K Chen B. The impact of obesity on adipocyte-derived extracellular vesicles. Cell Mol Life sciences: CMLS. (2021) 78:7275–88. doi: 10.1007/s00018-021-03973-w PMC8531905 34677643

[111] Ortega FJ Mercader JM Moreno-Navarrete JM Rovira O Guerra E Esteve E. Profiling of circulating microRNAs reveals common microRNAs linked to type 2 diabetes that change with insulin sensitization. Diabetes Care. (2014) 37:1375–83. doi: 10.2337/dc13-1847 24478399

[112] Chartoumpekis DV Zaravinos A Ziros PG Iskrenova RP Psyrogiannis AI Kyriazopoulou VE. Differential expression of microRNAs in adipose tissue after long-term high-fat diet-induced obesity in mice. PloS One. (2012) 7:e34872. doi: 10.1371/journal.pone.0034872 22496873 PMC3319598

[113] Santangelo A Imbrucè P Gardenghi B Belli L Agushi R Tamanini A. A microRNA signature from serum exosomes of patients with glioma as complementary diagnostic biomarker. J neuro-oncology. (2018) 136:51–62. doi: 10.1007/s11060-017-2639-x 29076001

[114] Ferrante SC Nadler EP Pillai DK Hubal MJ Wang Z Wang JM. Adipocyte-derived exosomal miRNAs: a novel mechanism for obesity-related disease. Pediatr Res. (2015) 77:447–54. doi: 10.1038/pr.2014.202 PMC4346410 25518011

[115] Ortega FJ Mercader JM Catalán V Moreno-Navarrete JM Pueyo N Sabater M. Targeting the circulating microRNA signature of obesity. Clin Chem. (2013) 59:781–92. doi: 10.1373/clinchem.2012.195776 23396142

[116] Can U Buyukinan M Yerlikaya FH. The investigation of circulating microRNAs associated with lipid metabolism in childhood obesity. Pediatr Obes. (2016) 11:228–34. doi: 10.1111/ijpo.12050 26223376

[117] Willeit P Skroblin P Moschen AR Yin X Kaudewitz D Zampetaki A. Circulating microRNA-122 is associated with the risk of new-onset metabolic syndrome and type 2 diabetes. Diabetes. (2017) 66:347–57. doi: 10.2337/db16-0731 PMC5248985 27899485

[118] Yu Y Du H Wei S Feng L Li J Yao F. Adipocyte-derived exosomal miR-27a induces insulin resistance in skeletal muscle through repression of PPARγ. Theranostics. (2018) 8:2171–88. doi: 10.7150/thno.22565 PMC5928879 29721071

[119] Wen D Qiao P Wang L. Circulating microRNA-223 as a potential biomarker for obesity. Obes Res Clin Pract. (2015) 9:398–404. doi: 10.1016/j.orcp.2015.01.006 25842981

[120] Geng L Lam KSL Xu A. The therapeutic potential of FGF21 in metabolic diseases: from bench to clinic. Nat Rev Endocrinology. (2020) 16:654–67. doi: 10.1038/s41574-020-0386-0 32764725

[121] Ying W Riopel M Bandyopadhyay G Dong Y Birmingham A Seo JB. Adipose tissue macrophage-derived exosomal miRNAs can modulate *in vivo* and *in vitro* insulin sensitivity. Cell. (2017) 171:372–84.e12. doi: 10.1016/j.cell.2017.08.035 28942920

[122] Zhu M Wei Y Geißler C Abschlag K Corbalán Campos J Hristov M. Hyperlipidemia-induced microRNA-155-5p improves β-cell function by targeting mafb. Diabetes. (2017) 66:3072–84. doi: 10.2337/db17-0313 28970282

[123] Fish JE Santoro MM Morton SU Yu S Yeh RF Wythe JD. miR-126 regulates angiogenic signaling and vascular integrity. Dev Cell. (2008) 15:272–84. doi: 10.1016/j.devcel.2008.07.008 PMC2604134 18694566

[124] Haneklaus M Gerlic M Kurowska-Stolarska M Rainey AA Pich D McInnes IB. Cutting edge: miR-223 and EBV miR-BART15 regulate the NLRP3 inflammasome and IL-1β production. J Immunol (Baltimore Md: 1950). (2012) 189:3795–9. doi: 10.4049/jimmunol.1200312 22984081

[125] Pescador N Pérez-Barba M Ibarra JM Corbatón A Martínez-Larrad MT Serrano-Ríos M. Serum circulating microRNA profiling for identification of potential type 2 diabetes and obesity biomarkers. PloS One. (2013) 8:e77251. doi: 10.1371/journal.pone.0077251 24204780 PMC3817315

[126] Hernández-Gómez KG Avila-Nava A González-Salazar LE Noriega LG Serralde-Zúñiga AE Guizar-Heredia R. Modulation of microRNAs and exosomal microRNAs after dietary interventions for obesity and insulin resistance: A narrative review. Metabolites. (2023) 13:1190. doi: 10.3390/metabo13121190 38132872 PMC10745452

[127] Kranendonk ME Visseren FL van Herwaarden JA Nolte-’t Hoen EN de Jager W Wauben MH. Effect of extracellular vesicles of human adipose tissue on insulin signaling in liver and muscle cells. Obes (Silver Spring Md). (2014) 22:2216–23. doi: 10.1002/oby.20847 25045057

[128] Sumaiya K Langford D Natarajaseenivasan K Shanmughapriya S. Macrophage migration inhibitory factor (MIF): A multifaceted cytokine regulated by genetic and physiological strategies. Pharmacol Ther. (2022) 233:108024. doi: 10.1016/j.pharmthera.2021.108024 34673115

[129] Lo JC Ljubicic S Leibiger B Kern M Leibiger IB Moede T. Adipsin is an adipokine that improves β cell function in diabetes. Cell. (2014) 158:41–53. doi: 10.1016/j.cell.2014.06.005 24995977 PMC4128197

[130] Wang GX Zhao XY Meng ZX Kern M Dietrich A Chen Z. The brown fat-enriched secreted factor Nrg4 preserves metabolic homeostasis through attenuation of hepatic lipogenesis. Nat Med. (2014) 20:1436–43. doi: 10.1038/nm.3713 PMC4257907 25401691

[131] Guo L Zhang P Chen Z Xia H Li S Zhang Y. Hepatic neuregulin 4 signaling defines an endocrine checkpoint for steatosis-to-NASH progression. J Clin Invest. (2017) 127:4449–61. doi: 10.1172/jci96324 PMC5707158 29106384

[132] Scheja L Heeren J. Metabolic interplay between white, beige, brown adipocytes and the liver. J hepatology. (2016) 64:1176–86. doi: 10.1016/j.jhep.2016.01.025 26829204

[133] Deng H Sun C Sun Y Li H Yang L Wu D. Lipid, protein, and microRNA composition within mesenchymal stem cell-derived exosomes. Cell Reporgram. (2018) 20:178–86. doi: 10.1089/cell.2017.0047 29782191

[134] Liang Q Zhong L Zhang J Wang Y Bornstein SR Triggle CR. FGF21 maintains glucose homeostasis by mediating the cross talk between liver and brain during prolonged fasting. Diabetes. (2014) 63:4064–75. doi: 10.2337/db14-0541 25024372

[135] Ruan CC Kong LR Chen XH Ma Y Pan XX Zhang ZB. A(2A) receptor activation attenuates hypertensive cardiac remodeling via promoting brown adipose tissue-derived FGF21. Cell Metab. (2018) 28:476–89.e5. doi: 10.1016/j.cmet.2018.06.013 30017353

[136] Yore MM Syed I Moraes-Vieira PM Zhang T Herman MA Homan EA. Discovery of a class of endogenous mammalian lipids with anti-diabetic and anti-inflammatory effects. Cell. (2014) 159:318–32. doi: 10.1016/j.cell.2014.09.035 PMC4260972 25303528

[137] Zhang Y Shi L Mei H Zhang J Zhu Y Han X. Inflamed macrophage microvesicles induce insulin resistance in human adipocytes. Nutr Metab. (2015) 12:21. doi: 10.1186/s12986-015-0016-3 PMC4462080 26064180

[138] Phu TA Ng M Vu NK Bouchareychas L Raffai RL. IL-4 polarized human macrophage exosomes control cardiometabolic inflammation and diabetes in obesity. Mol therapy: J Am Soc Gene Ther. (2022) 30:2274–97. doi: 10.1016/j.ymthe.2022.03.008 PMC9171286 35292359

[139] Heidarianpour A Keshvari M Shahidi S Zarei M. Modulation of GPC-4 and GPLD1 serum levels by improving glycemic indices in type 2 diabetes: Resistance training and hawthorn extract intervention. Heliyon. (2023) 9:e15537. doi: 10.1016/j.heliyon.2023.e15537 37151681 PMC10161711

[140] Reed JN Huang J Li Y Ma L Banka D Wabitsch M. Systems genetics analysis of human body fat distribution genes identifies adipocyte processes. Life Sci alliance. (2024) 7:e202402603. doi: 10.26508/lsa.202402603 38702075 PMC11068934

[141] Lee KY Yamamoto Y Boucher J Winnay JN Gesta S Cobb J. Shox2 is a molecular determinant of depot-specific adipocyte function. Proc Natl Acad Sci United States America. (2013) 110:11409–14. doi: 10.1073/pnas.1310331110 PMC3710789 23798383

[142] Arner P Kulyté A. MicroRNA regulatory networks in human adipose tissue and obesity. Nat Rev Endocrinol. (2015) 11:276–88. doi: 10.1038/nrendo.2015.25 25732520

[143] González-Sánchez GD Granados-López AJ López-Hernández Y Robles MJG López JA. miRNAs as interconnectors between obesity and cancer. Non-coding RNA. (2024) 10:24. doi: 10.3390/ncrna10020024 38668382 PMC11055034

[144] Liang D Li G. Pulling the trigger: Noncoding RNAs in white adipose tissue browning. Rev Endocrine Metab Disord. (2024) 25:399–420. doi: 10.1007/s11154-023-09866-6 38157150

[145] Mori MA Raghavan P Thomou T Boucher J Robida-Stubbs S Macotela Y. Role of microRNA processing in adipose tissue in stress defense and longevity. Cell Metab. (2012) 16:336–47. doi: 10.1016/j.cmet.2012.07.017 PMC3461823 22958919

[146] Mori MA Thomou T Boucher J Lee KY Lallukka S Kim JK. Altered miRNA processing disrupts brown/white adipocyte determination and associates with lipodystrophy. J Clin Invest. (2014) 124:3339–51. doi: 10.1172/jci73468 PMC4109560 24983316

[147] Li A Peng R Sun Y Liu H Peng H Zhang Z. LincRNA 1700020I14Rik alleviates cell proliferation and fibrosis in diabetic nephropathy via miR-34a-5p/Sirt1/HIF-1α signaling. Cell Death disease. (2018) 9:461. doi: 10.1038/s41419-018-0527-8 29700282 PMC5919933

[148] Xia W Chen H Chen D Ye Y Xie C Hou M. PD-1 inhibitor inducing exosomal miR-34a-5p expression mediates the cross talk between cardiomyocyte and macrophage in immune checkpoint inhibitor-related cardiac dysfunction. J Immunother cancer. (2020) 8:e001293. doi: 10.1136/jitc-2020-001293 33115945 PMC7594538

[149] Gao W Yuan LM Zhang Y Huang FZ Gao F Li J. miR-1246-overexpressing exosomes suppress UVB-induced photoaging via regulation of TGF-β/Smad and attenuation of MAPK/AP-1 pathway. Photochemical photobiological sciences: Off J Eur Photochem Assoc Eur Soc Photobiology. (2023) 22:135–46. doi: 10.1007/s43630-022-00304-1 36114328

[150] Jin J Shi Y Gong J Zhao L Li Y He Q. Exosome secreted from adipose-derived stem cells attenuates diabetic nephropathy by promoting autophagy flux and inhibiting apoptosis in podocyte. Stem Cell Res Ther. (2019) 10:95. doi: 10.1186/s13287-019-1177-1 30876481 PMC6419838

[151] Zhang H Guan M Townsend KL Huang TL An D Yan X. MicroRNA-455 regulates brown adipogenesis via a novel HIF1an-AMPK-PGC1α signaling network. EMBO Rep. (2015) 16:1378–93. doi: 10.15252/embr.201540837 PMC4766451 26303948

[152] Alvarez-Dominguez JR Bai Z Xu D Yuan B Lo KA Yoon MJ. *De novo* reconstruction of adipose tissue transcriptomes reveals long non-coding RNA regulators of brown adipocyte development. Cell Metab. (2015) 21:764–76. doi: 10.1016/j.cmet.2015.04.003 PMC4429916 25921091

[153] Zhang Y Song K Qi G Yan R Yang Y Li Y. Adipose-derived exosomal miR-210/92a cluster inhibits adipose browning via the FGFR-1 signaling pathway in high-altitude hypoxia. Sci Rep. (2020) 10:14390. doi: 10.1038/s41598-020-71345-8 32873843 PMC7463015

[154] Wang YC Li Y Wang XY Zhang D Zhang H Wu Q. Circulating miR-130b mediates metabolic crosstalk between fat and muscle in overweight/obesity. Diabetologia. (2013) 56:2275–85. doi: 10.1007/s00125-013-2996-8 23868745

[155] Wang YC Yao X Ma M Zhang H Wang H Zhao L. miR-130b inhibits proliferation and promotes differentiation in myocytes via targeting Sp1. J Mol Cell Biol. (2021) 13:422–32. doi: 10.1093/jmcb/mjab012 PMC8436675 33751053

[156] Piergentili R Marinelli E Cucinella G Lopez A Napoletano G Gullo G. miR-125 in breast cancer etiopathogenesis: an emerging role as a biomarker in differential diagnosis, regenerative medicine, and the challenges of personalized medicine. Non-coding RNA. (2024) 10:16. doi: 10.3390/ncrna10020016 38525735 PMC10961778

[157] Hartwig S De Filippo E Göddeke S Knebel B Kotzka J Al-Hasani H. Exosomal proteins constitute an essential part of the human adipose tissue secretome. Biochim Biophys Acta Proteins proteomics. (2019) 1867:140172. doi: 10.1016/j.bbapap.2018.11.009 30502511

[158] Lv J Yang S Lv M Lv J Sui Y Guo S. Protective roles of mesenchymal stem cells on skin photoaging: A narrative review. Tissue Cell. (2022) 76:101746. doi: 10.1016/j.tice.2022.101746 35182986

[159] Ndzie Noah ML Adzika GK Mprah R Adekunle AO Koda S Adu-Amankwaah J. Estrogen downregulates CD73/adenosine axis hyperactivity via adaptive modulation PI3K/Akt signaling to prevent myocarditis and arrhythmias during chronic catecholamines stress. Cell Commun Signal: CCS. (2023) 21:41. doi: 10.1186/s12964-023-01052-0 36823590 PMC9948346

[160] Gentile P Sterodimas A Pizzicannella J Calabrese C Garcovich S. Research progress on mesenchymal stem cells (MSCs), adipose-derived mesenchymal stem cells (AD-MSCs), drugs, and vaccines in inhibiting COVID-19 disease. Aging disease. (2020) 11:1191–201. doi: 10.14336/ad.2020.0711 PMC7505274 33014532

[161] Krasnodembskaya A Song Y Fang X Gupta N Serikov V Lee JW. Antibacterial effect of human mesenchymal stem cells is mediated in part from secretion of the antimicrobial peptide LL-37. Stem Cells (Dayton Ohio). (2010) 28:2229–38. doi: 10.1002/stem.544 PMC3293245 20945332

[162] Sutton MT Fletcher D Ghosh SK Weinberg A van Heeckeren R Kaur S. Antimicrobial properties of mesenchymal stem cells: therapeutic potential for cystic fibrosis infection, and treatment. Stem Cells Int. (2016) 2016:5303048. doi: 10.1155/2016/5303048 26925108 PMC4746399

[163] Pizzinat N Ong-Meang V Bourgailh-Tortosa F Blanzat M Perquis L Cussac D. Extracellular vesicles of MSCs and cardiomyoblasts are vehicles for lipid mediators. Biochimie. (2020) 178:69–80. doi: 10.1016/j.biochi.2020.07.013 32835733

[164] Morimoto K Shirata N Taketomi Y Tsuchiya S Segi-Nishida E Inazumi T. Prostaglandin E2-EP3 signaling induces inflammatory swelling by mast cell activation. J Immunol (Baltimore Md: 1950). (2014) 192:1130–7. doi: 10.4049/jimmunol.1300290 24342806

[165] Jiang MJ Chen YY Dai JJ Gu DN Mei Z Liu FR. Dying tumor cell-derived exosomal miR-194-5p potentiates survival and repopulation of tumor repopulating cells upon radiotherapy in pancreatic cancer. Mol cancer. (2020) 19:68. doi: 10.1186/s12943-020-01178-6 32228703 PMC7104536

[166] Halma MTJ Tuszynski JA Marik PE. Cancer metabolism as a therapeutic target and review of interventions. Nutrients. (2023) 15:4245. doi: 10.3390/nu15194245 37836529 PMC10574675

[167] Yu P Guo J Li J Shi X Xu N Jiang Y. lncRNA-H19 in fibroblasts promotes wound healing in diabetes. Diabetes. (2022) 71:1562–78. doi: 10.2337/db21-0724 35472819

[168] Lin JR Ding LL Xu L Huang J Zhang ZB Chen XH. Brown Adipocyte ADRB3 Mediates Cardioprotection via Suppressing Exosomal iNOS. Circ Res. (2022) 131:133–47. doi: 10.1161/circresaha.121.320470 35652349

[169] Liu Y Wang C Wei M Yang G Yuan L. Multifaceted roles of adipose tissue-derived exosomes in physiological and pathological conditions. Front Physiol. (2021) 12:669429. doi: 10.3389/fphys.2021.669429 33959041 PMC8093393

[170] Ren S Chen J Duscher D Liu Y Guo G Kang Y. Microvesicles from human adipose stem cells promote wound healing by optimizing cellular functions via AKT and ERK signaling pathways. Stem Cell Res Ther. (2019) 10:47. doi: 10.1186/s13287-019-1152-x 30704535 PMC6357421

[171] Woo CH Kim HK Jung GY Jung YJ Lee KS Yun YE. Small extracellular vesicles from human adipose-derived stem cells attenuate cartilage degeneration. J Extracell Vesic. (2020) 9:1735249. doi: 10.1080/20013078.2020.1735249 PMC7144299 32284824

[172] Son T Jeong I Park J Jun W Kim A Kim OK. Adipose tissue-derived exosomes contribute to obesity-associated liver diseases in long-term high-fat diet-fed mice, but not in short-term. Front Nutr. (2023) 10:1162992. doi: 10.3389/fnut.2023.1162992 37229466 PMC10203204

[173] Tao Y Chen W Xu H Xu J Yang H Luo X. Adipocyte-derived exosomal NOX4-mediated oxidative damage induces premature placental senescence in obese pregnancy. Int J Nanomed. (2023) 18:4705–26. doi: 10.2147/ijn.s419081 PMC10441661 37608820

